# Characterization of a caffeoyl-CoA *O*-methyltransferase-like enzyme involved in biosynthesis of polymethoxylated flavones in *Citrus reticulata*

**DOI:** 10.1093/jxb/eraa083

**Published:** 2020-03-17

**Authors:** Xiaojuan Liu, Chenning Zhao, Qin Gong, Yue Wang, Jinping Cao, Xian Li, Donald Grierson, Chongde Sun

**Affiliations:** 1 College of Agriculture & Biotechnology, Zhejiang University, Hangzhou, People’s Republic of China; 2 Zhejiang Provincial Key Laboratory of Horticultural Plant Integrative Biology, Zhejiang University, Hangzhou, People’s Republic of China; 3 The State Agriculture Ministry Laboratory of Horticultural Plant Growth, Development and Quality Improvement, Zhejiang University, Hangzhou, People’s Republic of China; 4 Division of Plant and Crop Sciences, School of Biosciences, University of Nottingham, Loughborough, UK; 5 Fondazione Edmund Mach, UK

**Keywords:** Citrus, flavonoids, fruit development, nobiletin, *O*-methyltransferase, polymethoxylated flavones

## Abstract

Polymethoxylated flavones (PMFs), which accumulate exclusively in fruit peel of citrus, play important physiological and pharmacological roles but the genetic basis for the methylation of flavonoids has not been fully elucidated in citrus. Here we characterize a caffeoyl-CoA *O*-methyltransferase-like enzyme, designated CrOMT1. The expression pattern of *CrOMT1* was highly correlated with the concentration of the three major PMFs in two different citrus fruit tissues during fruit maturation. Exposure of fruit to UV-B radiation sharply increased the level of *CrOMT1* transcripts and also led to the accumulation of three PMFs. The potential role of *CrOMT1* was studied by testing the catalytic activity of recombinant CrOMT1 with numerous possible substrates *in vitro*. The enzyme could most efficiently methylate flavones with neighboring hydroxy moieties, with high catalytic efficiencies found with 6-OH- and 8-OH-containing compounds, preferences that correspond precisely with the essential methylation sites involved in the synthesis of the three naturally occurring PMFs in *Citrus reticulata*. This indicates that CrOMT1 is capable of *in vitro* methylation reactions required to synthesize PMFs *in vivo*. Furthermore, transient overexpression of *CrOMT1* increased levels of the three major PMFs in fruit, indicating that CrOMT1 is likely to play an essential role in the biosynthesis of PMFs in citrus.

## Teaser

Identification of a *Citrus reticulata* CCoAOMT-like enzyme involved in biosynthesis of polymethoxylated flavones enables further investigation of these secondary metabolites in citrus fruit.

## Introduction

Flavonoids are a large class of polyphenols ubiquitous in plants. With various modifications (hydroxylation, acylation, glycosylation, methylation, etc.) to the basic skeleton of C_6_–C_3_–C_6_, flavonoids are generally categorized into six groups, namely flavonols, flavones, dihydroflavonols, dihydroflavones, isoflavones, and anthocyanins. Flavonoids with different structures contribute to pigment formation (Winkel-Shirley, 2001) and phytohormone signaling (Brunetti *et al.*, 2018) in plants, and they also play vital roles in defense against various biotic and abiotic stresses (Winkel-Shirley, 2001; Samanta *et al.*, 2011; Moore *et al.*, 2014; Nakabayashi *et al.*, 2014; Schulz *et al.*, 2015), including defense against herbivores and microorganisms, tolerance against drought and freezing, along with protection against UV-B irradiation (Li *et al.*, 1993; Landry *et al.*, 1995; Rozema *et al.*, 1997; Falcone Ferreyra *et al.*, 2010). Flavonoids have also gained increasing attention in recent years due to their beneficial effects on human health (Chen and Chen, 2013). It has been demonstrated that methylated flavonoids have greater biological activity than those that are unmethylated because methylation enhances their stability and oral bioavailability (Koirala *et al.*, 2016). Polymethoxylated flavones (PMFs) are enriched in the flavedo of citrus fruit, and the three major PMFs nobiletin, tangeretin, and 5-hydroxy-6,7,8,3',4'-pentamethoxyflavone (5-HPMF) have received increasing attention. These PMFs in citrus have been demonstrated to have potent effects as antioxidants, and as anti-cardiovascular disease and anti-cancer agents, and they also serve as nutraceutical supplements in the regulation of metabolic syndrome (Koirala *et al.*, 2016; Gao *et al.*, 2018). In addition to their pharmacological effects, PMFs are involved in plant defense against several important pathogens, including those causing common diseases in citrus: green mold and Huanglongbing (Berim and Gang, 2016). It is important to explore the biosynthesis of PMFs in citrus given their important physiological and pharmacological roles.

The hydroxy groups of flavonoids are methylated by *S*-adenosyl-l-methionine (SAM)-dependent *O*-methyltransferases (OMTs), which are critical enzymes for synthesis of PMFs. Plant OMTs are generally divided into two major subfamilies: the caffeic acid OMT (COMT) subfamily and the caffeoyl-CoA OMT (CCoAOMT) subfamily (Joshi and Chiang, 1998), based on the molecular weight and cation dependency of the proteins. Members of the COMT subfamily do not require cations to function and have a molecular weight ranging from 40 kDa to 43 kDa. Most OMTs involved in the methylation of flavonoids have been demonstrated to belong to this subfamily. Members of the CCoAOMT subfamily are smaller in size (26–30 kDa) and are usually cation dependent. CCoAOMT enzymes have been thought to use caffeoyl-CoA as a substrate and play important roles in the pathway of lignin biosynthesis (Kim *et al.*, 2010). However, a new Mg^2+^-dependent OMT with high sequence similarities to that of CCoAOMTs was identified from *Mesembryanthemum crystallinum* (Ibdah *et al.*, 2003). The enzyme, along with two highly homologous OMTs, exhibited substrate preferences for phenylpropanoids such as caffeic acid esters and flavonoids, and are clearly distinct from other CCoAOMTs, forming a new subgroup of CCoAOMT enzymes, designated PFOMT or CCoAOMT-like. Subsequently, several CCoAOMT-like proteins capable of methylating flavonoids were identified from different species, such as OsOMT15 and OsOMT17 from *Oryza sativa* subsp. *japonica* (Lee *et al.*, 2008) together with VpOMT4 and VpOMT5 from *Vanilla planifolia* (Widiez *et al.*, 2011), which are active against the *meta*-position of the flavonoid B-ring *in vitro*. Recombinant ObPFOMT-1 from *Ocimum basilicum* was shown to be a promiscuous enzyme with a preference for flavones favoring the 8-OH more than the 3'-OH (Berim and Gang, 2013). AtCCoAOMT7 identified from *Arabidopsis thaliana* displayed unusual *para*-methylation activity, compared with other reported CCoAOMTs. The recombinant enzyme methylated flavones and flavonols in the *meta*-position, while it methylated dihydroflavones and dihydroflavonols in the *para*-position, and the role of a single amino acid required for the conversion of *para* to *meta* was verified by incubating substrates with the CCoAOMT7 wild type and CCoAOMT7 single mutant, respectively (Wils *et al.*, 2013). In addition, CCoAOMT-like enzymes involved in the methylation of anthocyanins have been functionally characterized *in vivo* and *vitro*, including VvAOMT from *Vitis vinifera* (Hugueney *et al.*, 2009), SlAnthOMT from *Solanum lycopersicum* (Gomez Roldan *et al.*, 2014), and PsAOMT from *Paeonia suffruticosa* cv. ‘Gunpohden’ (Du *et al.*, 2015). The characterization of the above CCoAOMT-like enzymes indicated that this newly proposed subgroup involved in flavonoid methylation was likely to be present in many other species, making it of considerable importance to undertake further studies of this small group of plant enzymes, given their vital role in flavonoid methylation.

OMTs involved in flavonoid methylation have been functionally characterized from various plant species (Berim and Gang, 2016), including Arabidopsis, tomato, sweet basil, and peppermint. Recently, OMTs from citrus have been investigated and *in silico* analysis revealed 58 *OMT* genes in sweet orange (Liu *et al.*, 2016). Nobiletin and tangeretin are examples of methylated flavonoids with significant bioactivity found exclusively in citrus species. Phylogenetic analysis with functionally characterized OMTs from other plants suggested that 27 of these genes could be involved in *O*-methylation of flavonoids in citrus, and their expression profiles were analyzed in different tissues and fruit developmental stages (Liu *et al.*, 2016). Five *OMT* genes belonging to the *COMT* subfamily were isolated from *Citrus depressa* and one, termed *CdOMT5*, was successfully expressed in *Escherichia coli* (Itoh *et al.*, 2016). Recombinant CdOMT5 exhibited broad substrate regioselectivity for the 3, 5, 6, and 7 positions of flavones, and bioconversion of quercetin into its 3,3',5,7 tetra-methyl ether was accomplished in *E. coli* overexpressing *CdFOMT5*. This multifunctional property implied the possible role of the enzyme in the biosynthesis of PMFs. The expression patterns of this gene and the catalytic activity of the protein with different substrates need to be analyzed to confirm this hypothesis.

In this study, we describe the identification and biochemical characterization of CrOMT1, a CCoAOMT-like enzyme involved in the biosynthesis of PMFs in citrus, its expression in the peel of ‘Ougan’ fruit (*Citrus reticulata* cv. *Suavissima*), and the correlation between its expression profile and PMF accumulation. Recombinant CrOMT1 shows a remarkable preference for analogs of potential PMF-type substrates, and transient overexpression assays in the peel of citrus confirmed the role of the enzyme in enhancing accumulation of PMFs. This is the first identification of a citrus CCoAOMT-like enzyme responsible for PMF biosynthesis both *in vitro* and *vivo*, and opens the way for investigation of biosynthesis of PMFs in citrus.

## Materials and methods

### Chemicals sources

Acetonitrile and methanol of HPLC grade were purchased from Sigma-Aldrich (St. Louis, MO, USA). Quercetin 3-methyl ether, eriodictyol, ampelopsin (dihydromyricetin), taxifolin (dihydroquercetin), aromadendrin (dihydrokaempferol), tamarixetin, quercetin 3,3'-dimethyl ether, isokaempferide, chrysoeriol, homoeriodictyol, and isoferulic acid were purchased from BioBioPha Co., Ltd (Kunming, China), and oroxylin A was purchased from Shanghai Yuanye Bio-Technology Co., Ltd (Shanghai, China). Laricitrin, syringetin, and tricetin were from Extrasynthese (Lyon, France), and tricin was from ChromaDex (Irvine, CA, USA). Quercetin, isorhamnetin, kaempferol, luteolin, baicalein, eriocitrin, SAM, and formic acid for HPLC were obtained from Aladdin (Shanghai, China). Myricetin, apigenin, and 7,8-dihydroxyflavone hydrate were purchased from TCI (Shanghai, China). Caffeic acid, naringenin, hesperetin, neoeriocitrin, and ferulic acid were also purchased from Sigma-Aldrich.

### Plant materials and treatment

Fruits of the ‘Ougan’ cultivar (*Citrus reticulata* cv. *Suavissima*) were sampled from Wenzhou City, China, at eight progressive maturation stages: S1, 30 days after flowering (DAF); S2, 60 DAF; S3, 80 DAF; S4, 100 DAF; S5, 120 DAF; S6, 140 DAF; S7, 170 DAF; and S8, 200 DAF. Samples were separated into four biological replicates, and the peels were divided into flavedo and albedo parts (albedo could be separated from the peel at S5, S6, S7, and S8).

‘Ougan’ fruits at S7 were also subjected to UV light irradiation. A total of five replicates were performed in UV-B treatment. For each fruit, the side irradiated by UV-B light and the unexposed side were set as the treatment group and control group, respectively. The procedure was carried out with UV-B (280–315 nm) light of 50 μW cm^–2^ at a constant temperature of 10 °C, and the flavedo of fruits was sampled 24 h and 48 h after irradiation. Samples of ‘Ougan’ fruit developmental stages and UV-B treatments were frozen immediately in liquid nitrogen and stored at –80 °C for subsequent analysis.

Fruits of the ‘Ougan’ cultivar, at 160 DAF, randomly sampled from Hangzhou City, China were used to perform transient expression analysis once detached from the tree, as described later.

### RNA isolation, RNA sequencing, and qRT–PCR

Total RNA of samples (‘Ougan’ developmental stages and UV-B treatment) was extracted according to the method described by Kiefer *et al.* (2000), and treated with gDNA Eraser to remove genomic DNA (gDNA) contamination via a PrimeScript™ RT reagent Kit (Takara, Dalian, China). RNA sequencing (RNA-Seq) was performed to analyze the expression pattern of *OMT* genes in flavedo of fruit peel during ‘Ougan’ developmental stages (S1, S3, S5, and S7) using an Illumina HiSeq sequencer (Shanghai Majorbio Bio-pharm Technology, Ltd, Shanghai, China). For RNA samples extracted from fruit flavedo after UV-B treatment, RNA-Seq (Quantification) was also conducted using a BGISEQ-500RS sequencer (Shenzhen Huada Gene Science and Technology Service Co., Ltd, Shenzhen, China). The gene expression level determined by RNA-Seq was evaluated by FPKM (fragments per kilobase of exon per million fragments mapped) values. All the reads were aligned to the *Citrus clementina* reference genome (http://www.citrusgenomedb.org/species/clementina/genome1.0) (Wu *et al.*, 2014).

Real-time quantitative reverse transcription–PCR (qRT–PCR) was performed to verify the temporal and spatial expression pattern of *CrOMT1* in ‘Ougan’ fruit. Briefly, total RNA was extracted and used to synthesize cDNA as described above. Gene-specific oligonucleotide primers were designed by NCBI Primer-BLAST (https://www.ncbi.nlm.nih.gov/tools/primer-blast/) with their gene specificity checked by melting curve and PCR re-sequencing. Citrus β-actin served as a housekeeping gene for normalization (Pillitteri *et al.*, 2004) and ∆Ct was used to calculate the relative abundance of gene transcripts. Real-time PCRs were performed as described before (Liu *et al.*, 2017). Gene-specific primers mentioned above are listed in Supplementary Table S1 at *JXB* online.

### Phylogenetic analysis and sequence alignment

The deduced amino acid sequences of 55 identified *OMT* genes expressed in the flavedo of ‘Ougan’ fruit were aligned with various currently known plant OMT members, including the COMT subfamily and CCoAOMT subfamily, using the ClustalW bundled in MEGA X software (Mega Software, State College, PA, USA) (Kumar *et al.*, 2018). Construction of an unrooted phylogenetic tree was based on the Neighbor–Joining statistical method (Saitou and Nei, 1987) and was tested with 1000 bootstrap replicates (Felsenstein, 1985). iTOL software (https://itol.embl.de/) was used to display and annotate the phylogenetic tree. UniProt entries of proteins from other species used above are given in Supplementary Table S2.

Sequences of members belonging to the CCoAOMT subfamily were aligned using structural information via Expresso bundled in T-Coffee (Notredame *et al.*, 2000), and key features among the sequences were deciphered through ESPript 3.0 web server (Robert and Gouet, 2014). Sequence identity of CrOMT1 with other identified PFOMTs was calculated using local BLASTP.

### Isolation, cloning, and heterologous expression of *CrOMT1*

The *CrOMT1* gene was isolated based on the *C. clementina* reference genome. The full coding sequence (CDS) of *CrOMT1* was amplified using the primers described in Supplementary Table S1 and then subcloned into the pET32a vector (without a stop codon) followed by transfer to *E. coli* strain BL21(DE3)pLysS (Promega, Madison, WI, USA) for expression. Transformants carrying the expression plasmid *CrOMT1*-PET were incubated to OD_600_=0.6 in Luria–Bertani medium containing 0.1 g l^–1^ ampicillin at 37 °C, followed by the addition of isopropyl-β-d-thiogalactopyranoside (IPTG) to a final concentration of 1 mM and then the incubation was carried out for 24 h at 18 °C. After induction, the bacterial cells were collected (4000 rpm, 4 °C, 10 min) and resuspended in 1× PBS buffer. The clarified supernatant of cells disrupted by sonication was obtained by centrifugation at 4 °C and the corresponding recombinant proteins were purified using a HisTALON™ Gravity Columns Purification Kit (Takara, Dalian, China) according to the user manual. Collected fractions were transferred into storage buffer (50 mM Tris–HCl, pH 8.0, 10% glycerol, and 2 mM DTT) through a PD-10 desalting column (GE Healthcare, Uppsala, Sweden), and stored at –80 °C for further analysis. Protein concentrations were determined via the Modified BCA Protein Assay Kit (Sangon Biotech, Shanghai, China).

### Enzyme assays and kinetics

Enzyme reactions for recombinant CrOMT1-pET were performed with different phenolic substrates, including flavonoids and caffeic acid. Reaction mixtures consisted of Tris–HCl buffer (50 mM, pH 8.0), 1 mM SAM, 200 μM phenolic substrates, and 25 μl of purified protein in a final volume of 200 μl. Assays were incubated in 37 °C for 2 h and extracted twice with an equal volume of ethyl acetate. The upper organic phase was dried and resolved in methanol, and then filtered through a 0.22 μm filter for HPLC or LC-MS.

The effect of pH on CrOMT1 was estimated in Tris–HCl buffer for pH 7.0–9.0 and potassium phosphate buffer for pH 5.5–8.0. Temperature ranging from 25 °C to 80 °C was set to determine the optimum temperature for CrOMT1. Both assays were performed with luteolin.

Relative activity of various phenolic substrates was measured via MTase-Glo™ Methyltransferase Assay (Promega). The product of SAM in a methylation reaction was converted to ADP and then the ADP was converted to ATP, which could be detected by a plate-reading luminometer, and relative luminescence was correlated to SAH (*S*-adenosylhomocysteine) concentration by generating an SAH standard curve. In detail, assays were processed with phenolic substrate concentration at 12.5 μM, SAM at 250 μM, and appropriate dilutions of the purified enzyme in Tris–HCl buffer (pH 8.0). After 30 min of incubation at 37 °C, the reaction was stopped by adding 0.5% TFA (trifluoroacetic acid), followed by estimation of SAH by luminescence.

For kinetic analysis of the recombinant proteins, a serial dilution of phenolic substrates along with 2 mM SAM as methyl donor was used to conduct enzyme assays in the same conditions as mentioned above. The kinetic parameters *K*_m_ and *K*_cat_ were estimated by non-linear regression curve fit using Michaelis–Menten bundled in GraphPad Prism version 7 (GraphPad Software, San Diego, CA, USA).

### Transient overexpression of *CrOMT1* in ‘Ougan’ fruit peel

The full-length CDS of *CrOMT1* was cloned into the pBI121 expression vector containing the *Cauliflower mosaic virus 35S* promoter. The recombinant vector was transformed into *Agrobacterium* (EHA105). *CrOMT1* transient overexpression in ‘Ougan’ fruit peel was conducted by *Agrobacterium tumefaciens*-mediated infiltration as described previously (Yin *et al.*, 2016). Specifically, each fruit was set as a replicate, and seven fruits were used in transient analysis. Two points on the equatorial face on opposite sides of each fruit were selected, and one was injected with *Agrobacterium* suspension containing the target gene and the other with corresponding empty vector (pBI121) acting as a control. For each injection site, 1 ml of the corresponding *Agrobacterium* suspension (OD_600_=0.75) was used. Contents of PMFs and transcript levels of *CrOMT1* were measured 5 d after infiltration by cutting out a region of tissue ~3 cm in diameter surrounding the injection site. Primers used in the construction of *CrOMT1*-pBI121 and for qRT–PCR are listed in Supplementary Table S1.

### Metabolite analysis

Determination of flavonoid composition was carried out as described by Zhang *et al.* (2012) with minor modifications. Different tissues were ground under liquid nitrogen and up to 500 mg of frozen powder was extracted in 3 ml of 80% ethanol with sonication for 30 min; then the supernatants were obtained by centrifugation at 4000 rpm for 10 min. The samples were extracted three times and the supernatant fractions (~9 ml) were combined. The ethanol-based extract was filtered through a 0.22 μm membrane prior to HPLC analysis.

### HPLC, mass spectrometry, and NMR spectroscopy

For HPLC, an Agilent (Agilent Technologies, Santa Clara, CA, USA) 1260 HPLC system equipped with a quaternary pump and a VWD detector was used in all the chromatographic experiments. Samples were separated at room temperature by a Sunfire C18 ODS column (4.6×250 mm, 5 μm, Waters Corp., Milford, MA, USA), at a flow rate of 1 ml min^–1^. The mobile phases were acetonitrile (A) and 0.1% formic acid–water (B) with a linear gradient program as follows, 0/20, 5/20, 10/27, 15/27, 25/40, 35/60, 40/80, 42/100, 45/20, and 50/20 (min/A%). The injection volume of samples was 10 μl. Detection was by UV at 280 nm (dihydroflavones, dihydroflavonols, and caffeic acid), 330 nm (nobiletin, tangeretin, and 5-HPMF), and 350 nm (flavones and flavonols).

MS analysis was performed using an AB TripleTOF 5600plus System (AB SCIEX, Framingham, MA, USA). MS spectra were obtained in negative ion mode or positive ion mode (ESI). The exact mass calibration was measured automatically before each analysis employing the Automated Calibration Delivery System.

For NMR spectroscopy, a reaction mixture of 7,8-dihydroxyflavone methylated by CrOMT1 was extracted twice with an equal volume of ethyl acetate. The upper organic phase was dried using a rotary evaporator and then resolved in methanol for subsequent separation and collection by means of semi-preparative HPLC. Collected samples were completely dried and then dissolved in DMSO-d6 (bis-trideuteriomethyl sulfoxide) (99.9 atoms % D) containing 0.03% TMS (tetramethylsilane). TMS standard was used for standardization of chemical shifts in NMR solvents. Proton (^1^H) NMR (500 MHz) and carbon (^13^C) NMR (125 MHz) spectra were recorded on a Bruker Avance III 500M spectrometer (Bruker, Switzerland).

### Statistical analyses

All data were obtained with at least three replicates and are presented as mean values and SEs. Graphics were drawn using OriginPro 2019 Learning Edition (Microcal Software Inc., Northampton, MA, USA) and GraphPad Prism version 7 (GraphPad Software, San Diego, CA, USA). Structural formulae were produced using ChemBioDraw Ultra 12.0 (PerkinElmer Informatics, Waltham, MA, USA).

## Results

### PMF accumulation in ‘Ougan’ peel during fruit maturation and in response to UV-B treatment

**Fig. 1.**
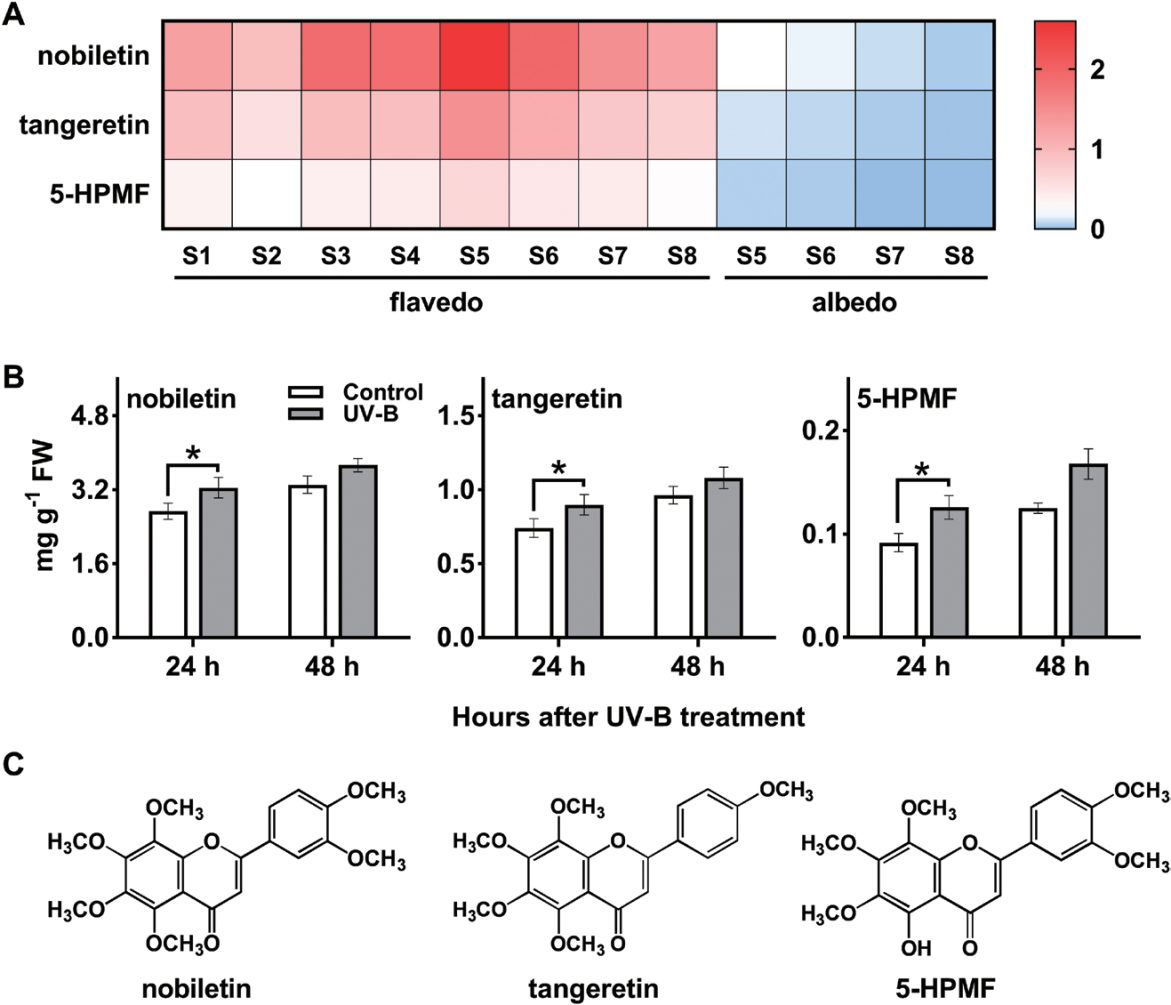
Dynamic changes of PMF levels during ‘Ougan’ fruit maturation and in response to UV-B irradiation. (A) Heatmap display of the three most abundant PMFs in different tissues. (Original data are shown in Supplementary Table S5.) 5-HPMF, 5-hydroxy-6,7,8,3',4'-pentamethoxyflavone; color ranges represent contents of flavonoids and values are mg g^–1^ FW. (B) Changes of the three most abundant PMFs in the flavedo of ‘Ougan’ fruit under UV-B irradiation. Values are means ±SE (*n*=5). *P<0.05 (right-tailed paired *t*-test, *n*=5). (C) Structural formulae of the three most abundant PMFs.

PMFs were investigated in ‘Ougan’ fruit at eight different developmental stages. The peel of ‘Ougan’ fruit, which is known to accumulate abundant contents of PMFs (Wang *et al.*, 2017), was used to determine detailed flavonoid metabolites. The three most abundant PMFs detected were nobiletin, tangeretin, and 5-HPMF according to the retention time of corresponding authentic standards and published literature (Wang *et al.*, 2017). Nobiletin, which has six methoxy groups, was the most abundant PMF detected in citrus peel (Fig. 1). High contents of PMFs were observed in flavedo samples and peaked at S5; while they were barely detected in albedo tissue (Fig. 1A).

Significant increases were observed in the accumulation of nobiletin, tangeretin, and 5-HPMF in ‘Ougan’ fruit flavedo after irradiation for 24 h with UV-B, compared with the control group. This was maintained after irradiation for 48 h, although there was no significant further increase (Fig. 1B).

### Isolation of a candidate *OMT* cDNA from ‘Ougan’ fruit peel

To identify putative *OMT* genes involved in PMF biosynthesis in ‘Ougan’ fruit, expression profiles of *OMT* genes in the flavedo of ‘Ougan’ fruit during developmental stages (S1, S3, S5, and S7) were obtained from RNA-Seq data. On the basis of gene annotations in the *C. clementina* reference genome and subsequent conserved domain search (https://www.ncbi.nlm.nih.gov/Structure/cdd/wrpsb.cgi), a total of 55 putative *OMT* genes were found to be expressed in the flavedo of ‘Ougan’ fruit during maturation (Supplementary Table S3).

**Fig. 2.**
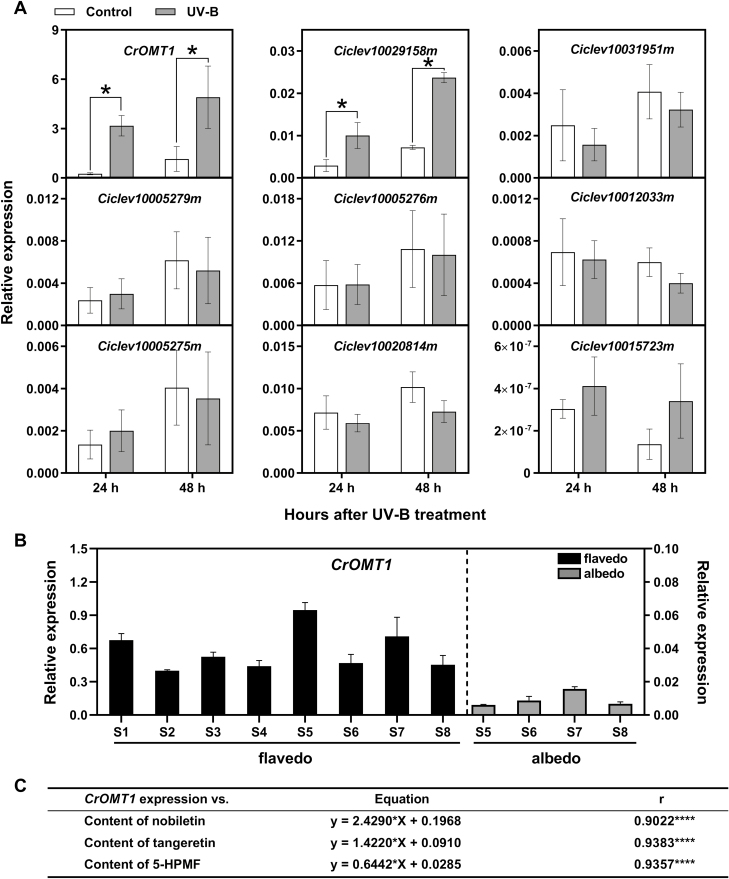
Comparison of *CrOMT1* transcript levels and accumulation of PMFs. (A) Transcript levels of *CrOMT1* and another eight potential *OMT* genes in response to UV-B irradiation. Values are means ±SE (*n*=5). *P<0.05 (right-tailed paired *t*-test, *n*=5). (B) Relative expression of *CrOMT1* in different tissues and during ‘Ougan’ fruit maturation. Values are means ±SE (*n*=4). (C) The correlation between transcript levels of *CrOMT1* and the contents of PMFs during ‘Ougan’ fruit maturation.

To aid selection of potential *OMT* genes involved in PMF accumulation, correlation coefficients (Pearson *r*) between *OMT* gene transcript levels and PMF contents were calculated. Only nine of the 55 *OMT* genes were considered to have the potential to be involved in the synthesis of PMFs during fruit maturation using the following selection criteria: first, a correlation coefficient >0.7 for expression versus concentration of at least one PMF (nobiletin, tangeretin, or 5-HPMF); and, secondly, expression level of FPKM >50 for at least one developmental stage (Supplementary Table S3). Of the nine *OMT* genes that fulfilled these criteria, only one (*Ciclev10026344m*, termed *CrOMT1*) had the highest transcript levels (~12-fold and ~14-fold higher) in ‘Ougan’ flavedo irradiated by UV-B for 24 h and 48 h, compared with the control group (Fig. 2A). The expression pattern of *CrOMT1* in the flavedo and albedo of ‘Ougan’ fruit at eight stages was verified by qRT–PCR. *CrOMT1* was specifically expressed in the flavedo, and the transcript abundance was highest at the S5 stage (relative expression level=0.95). The transcript levels of *CrOMT1* were much lower in the albedo, and the relative expression levels at the S5, S6, S7, and S8 stages were 0.006, 0.009, 0.016, and 0.007, respectively (Fig. 2B). The expression levels of *CrOMT1* were highly correlated with the accumulation of PMFs in different developmental stages and tissues (Fig. 2C). These results suggested that *CrOMT1* might potentially be involved in the synthesis of PMFs.

### *In silico* analysis of CrOMT1

The cDNA encoding CrOMT1 was obtained from a cDNA library of ‘Ougan’ fruit peel using gene-specific primers. The ORF of *CrOMT1* was 750 bp, coding for 249 amino acids. The calculated isoelectric point and molecular mass of the predicted amino acid sequence was 5.13 and 28.12 kDa, respectively (ExPASy Compute pI/Mw Tool).

**Fig. 3.**
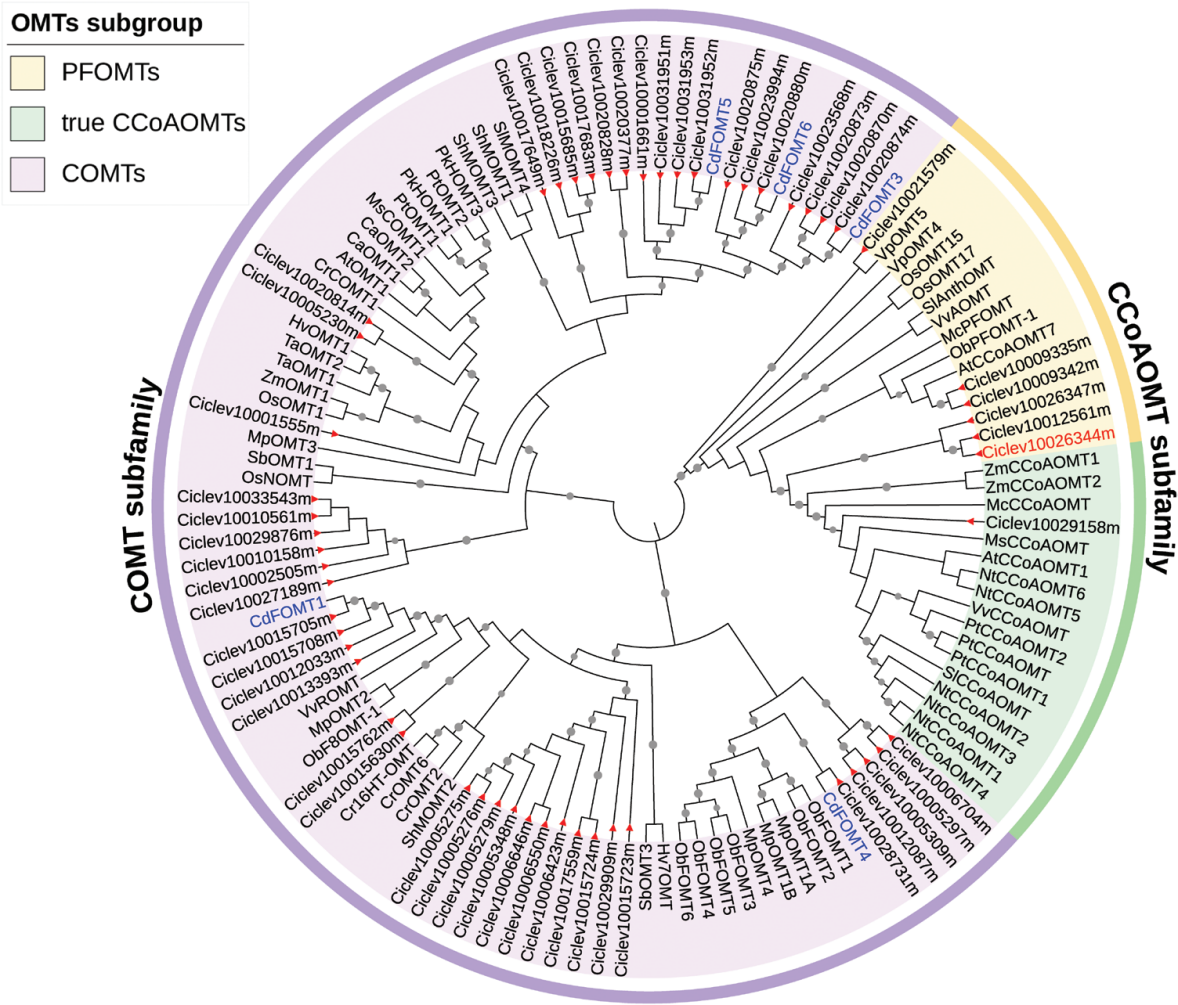
Phylogenetic analysis of OMTs detected in the flavedo of ‘Ougan’ fruit with other plant OMTs. The evolutionary history was constructed using the Neighbor–Joining method and the tree was tested with 1000 bootstrap replicates. Bootstrap values are only displayed if the values are >70%, and each value is visualized using a gray circle, whose size represents the corresponding metadata value, in the middle of each branch. Two main clusters (COMT subfamily and CCoAOMT subfamily) are indicated in the phylogenetic tree. COMT subfamily, indicated in purple. PFOMTs and true CCoAOMTs belong to the CCoAOMT subfamily, indicated in yellow and green, respectively. OMTs from ‘Ougan’ are indicated with a red triangle at the end of each branch. CdFOMTs from *Citrus depressa* (Itoh *et al.*, 2016) are indicated in blue. CrOMT1 (Ciclev10026344m) in this study is indicated in red.

CrOMT1 contained a conserved domain characteristic of the AdoMet_Mtases superfamily (accession: cl17173) and was clustered with members of the CCoAOMT subfamily on the phylogenetic tree (Fig. 3). Generally, members of this family have been demonstrated to be specifically active with phenylpropanoid-CoA esters, and function as key enzymes in the lignin biosynthesis pathway (Kim *et al.*, 2010). However, a new subgroup of this subfamily, named PFOMT or CCoAOMT-like, was proposed recently (Ibdah *et al.*, 2003). PFOMT methylates a wide range of metabolites and phenylpropanoids, with caffeic acid esters and flavonoids as preferred substrates. Consideration of the wide differences in substrate spectrum led to the CCoAOMT subfamily being divided into two subgroups: PFOMTs and true CCoAOMTs (Ibdah *et al.*, 2003). Interestingly, CrOMT1 was clustered close to other PFOMTs (Fig. 3), with >50% amino acid sequence identity to PFOMTs from other plants, with the exception of VpOMT5 from *Vanilla planifolia*, which has a chloroplast transit peptide at the N-terminus (Widiez *et al.*, 2011) (Supplementary Table S4). This close phylogenetic relationship with PFOMTs suggested that CrOMT1 was likely to accept flavonoids as favorable substrates (Fig. 3).

Amino acid sequences of enzymes in the PFOMT subgroup were aligned with several members of the true CCoAOMT subgroup and compared using secondary structural information. The substrate specificity of the CCoAOMT subfamily is believed to be influenced by two regions: the N-terminus and an insertion loop near the C-terminus (Ibdah *et al.*, 2003; Kopycki *et al.*, 2008) (Supplementary Fig. S1). Two unique Tyr residues, boxed in red in Supplementary Fig. S1, were fully conserved within the true CCoAOMTs, and the two Tyr residues have been implicated in caffeoyl-CoA binding (Walker *et al.*, 2016). However, they were substituted by distinct residues within the PFOMTs, and in CrOMT1 they were also substituted by Leu and Trp, respectively. This indicated that CrOMT1 was likely to be active on substrates other than caffeoyl-CoA.

### Regioselectivity of recombinant CrOMT1

The CrOMT1 enzyme fused with two histidine tags was expressed in *E. coli*, and the recombinant protein formed a band at ~46 kDa on SDS–PAGE, which is consistent with the predicted mass of 46.89 kDa (Supplementary Fig. S2). In order to explore the substrate specificity of CrOMT1, the recombinant enzyme was tested with numerous phenolic substrates representing three different flavonoid subclasses, flavonols, flavones, and dihydroflavones, and caffeic acid, assayed with SAM as the methyl donor. Identification of methylated products was conducted by retention time and MS/MS data compared with the authentic compound. For the product without commercial standards, methylation sites were inferred from the obtained results by similar substrates, published literature, or NMR spectroscopy. (Table 1; Supplementary Figs S3, S4). For better understanding of the methylation sites for each substrate, schematic diagrams with chemical structures are shown in Supplementary Fig. S5.

**Table 1.** Compounds identified by LC-MS analysis of reaction products produced by recombinant CrOMT1

Substrate	Retention time (min)	Theoretical [M+H]^+^	Product	Retention time [min]	Theoretical [M+H]^+^	Observed [M+H]^+^	Methylated position
Flavonols							
Quercetin	23.251	303.0505	Isorhamnetin	28.985	317.0661	317.0644	3'^∆^
			Tamarixetin	29.208	317.0661	317.0651	4'^∆^
			Quercetin 3',4'-dimethyl ether	30.303	331.0818	331.0819	3',4'
Quercetin 3-methyl ether	25.056	315.0505^○^	Quercetin 3,3'-dimethyl ether	30.420	329.0661^○^	329.0673^○^	3'^∆^
Myricetin	15.453	319.0454	Laricitrin	22.765	333.0610	333.0597	3'^∆^
			Syringetin	28.189	347.0767	347.0754	3',5'^∆^
			Myricetin dimethyl ether	29.960	347.0767	347.0749	3',4'
Kaempferol	28.497	287.0556	Isokaempferide	29.856	301.0712	301.0718	3^∆^
Flavones							
Luteolin	22.967	285.0399^○^	Chrysoeriol	28.319	299.0556^○^	299.0564^○^	3'^∆^
Tricetin	16.739	303.0505	Tricetin mono methyl ether	22.735	317.0661	317.0663	3'
			Tricetin mono methyl ether	23.193	317.0661	317.0659	4'
			Tricin	27.212	331.0818	331.0821	3',5'^∆^
			Tricetin dimethyl ether	29.279	331.0818	331.0825	3',4'
Apigenin	27.514		ND				
Baicalein	28.778	271.0606	Oroxylin A	35.379	285.0763	285.0774	6^∆^
7,8-Dihydroxyflavone	21.403	255.0657	7-Hydroxy-8-methoxyflavone	28.918	269.0814	269.0806	8^∆^
Dihydroflavones							
Eriodictyol	22.044	287.0556^○^	Homoeriodictyol	28.072	301.0712^○^	301.0706^○^	3'^∆^
			Hesperetin	28.765	301.0712^○^	301.0722^○^	4'^∆^
Eriocitrin	8.004	595.1663^○^	Homoeriodictyol 7-*O*-rutinoside	12.336	609.1819^○^	609.1848^○^	3'
			Hesperidin	13.144	609.1819^○^	609.1847^○^	4'^∆^
Neoeriocitrin	9.517	595.1663^○^	Homoeriodictyol 7-*O*-neohesperidoside	13.240	609.1819^○^	609.1838^○^	3'
			Neohesperidin	13.941	609.1819^○^	609.1845^○^	4'^∆^
Naringenin	27.975		ND				
Hesperetin	29.059		ND				

ND, not detectable; theoretical [M+H]^+^, exact mass of compound calculated by ChemBioDraw in positive mode; observed [M+H]^+^, exact mass of compound observed in MS/MS data. Values with a circle represent corresponding exact mass [M-H]^–^ in negative mode. Values with a triangle represent methylation position identified by MS/MS in comparison with that of the authentic standard or by NMR.

**Fig. 4.**
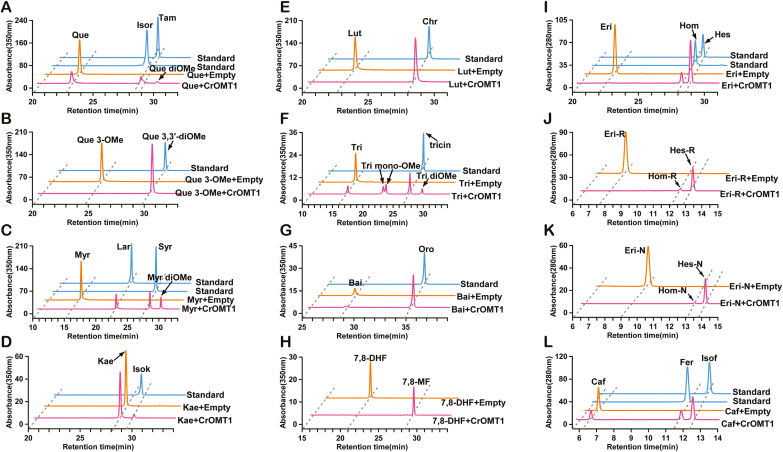
HPLC chromatograms of methylation of flavonols, flavones, dihydroflavones, and caffeic acid by CrOMT1 *in vitro*. Substrates utilized by recombinant CrOMT1 are indicated in magenta. Substrates incubated with the empty vector (pET32a) are indicated in orange. Authentic compounds of methylated products are indicated in blue. (A–D) Flavonols which acted as substrates: Que, quercetin; Isor, isorhamnetin; Tam, tamarixetin; Que diOMe, quercetin dimethyl ether; Que 3-OMe, quercetin 3-methyl ether; Que 3,3'-diOMe, quercetin 3,3'-dimethyl ether; Myr, myricetin; Lar, laricitrin; Syr, syringetin; Myr diOMe, myricetin dimethyl ether; Kae, kaempferol; Isok, isokaempferide. (E–H) Flavones which served as substrates: Lut, luteolin; Chr, chrysoeriol; Tri, tricetin; Tri mono-OMe, tricetin mono methyl ether; Tri diOMe, tricetin dimethyl ether; Bai, baicalein; Oro, oroxylin A, 7,8-DHF, 7,8-dihydroxyflavone; 7,8-MF, 7-hydroxy-8-methoxyflavone. (I–K) Dihydroflavones methylated by CrOMT1: Eri, eriodictyol; Hom, homoeriodictyol, Hes, hesperetin; Eri with R and N indicate their corresponding 7-*O*-rutinoside and 7-*O*-neohesperidoside, respectively. (L) Caffeic acid methylated by CrOMT1: Caf, caffeic acid; Fer, ferulic acid; Isof, isoferulic acid. MS/MS data of products and their authentic compounds are indicated in Supplementary Fig. S3.

The flavonols quercetin, quercetin 3-methyl ether, myricetin, and kaempferol were tested (Fig. 4A–D). Quercetin was converted to its 3'-methyl ether (isorhamnetin), 4'-methyl ether (tamarixetin), and eventually formed the di-methyl ether (quercetin 3',4'-dimethyl ether), whose structure was deduced from the time-dependent methylation reaction and MS (Fig. 4A; Supplementary Figs S3, S6). The reaction kinetics showed that the two mono-methyl ethers could be produced by CrOMT1 *in vitro*, but the amount of di-methyl ether increased while the two mono-methyl products decreased during extended reaction times (Supplementary Fig. S6). This indicated that quercetin could be converted into the final product quercetin 3',4'-dimethyl ether by CrOMT1 *in vitro*. To our knowledge, this is the first report that CCoAOMT-like enzyme could sequentially catalyze vicinal hydroxy groups on the B-ring of quercetin. The methylation site was restricted to the 3' position with quercetin 3-methyl ether as substrate, yielding a single product, its 3'-*O*-methylation product (quercetin 3,3'-dimethyl ether) (Fig. 4B). Three products were obtained with myricetin as substrate. Two were identified as myricetin 3'-methyl ether (laricitrin) and myricetin 3',5'-dimethyl ether (syringetin), and the third peak was myricetin di-methyl ether according to MS (Fig. 4C; Supplementary Fig. S3). Quercetin and myricetin have similar structures (the latter has an additional hydroxy group on the B-ring); therefore, methylation might be expected at the 3' and 4' positions, but so far this has not been confirmed. With kaempferol, which carries a single hydroxy group on the B-ring, only a tiny peak, representing 3-methylated kaempferol (isokaempferide), was detected (Fig. 4D).

Luteolin, tricetin, apigenin, baicalein, and 7,8-dihydroxyflavone, which are classified as flavones, were incubated with recombinant CrOMT1 separately (Fig. 4E–H). Luteolin, which lacks 3-OH compared with quercetin, was converted into a single product, chrysoeriol (luteolin 3'-methyl ether) (Fig. 4E). With tricetin as substrate, four products were produced by CrOMT1. One was identified as tricin (tricetin 3',5'-dimethyl ether) and the other three were two tricetin mono-methyl ethers and one di-methyl ether identified by MS (Fig. 4F; Supplementary Fig. S3). Production of four methylation products is similar to the results obtained with ObPFOMT-1 from *Ocimum basilicum*, which also yielded four products, two mono-methylated and two di-methylated, when incubated with tricetin (Berim and Gang, 2013). Therefore, the other three products were probably 3'-methyl ether, 4'-methy ether, and 3',4'-dimethyl ether by comparison with tricetin derivatives produced by ObPFOMT-1. Apigenin, lacking vicinal dihydroxy groups on the B-ring, could not be methylated by the enzyme (Table 1; Supplementary Fig. S5). Baicalein, which has three hydroxy groups in positions 5, 6, and 7 on the A-ring, was converted into its 6-methyl ether (oroxylin A) (Fig. 4G). With 7,8-dihydroxyflavone as substrate, a single product was identified as 7-hydroxy-8-methoxyflavone by MS, ^1^H NMR, and ^13^C NMR spectra. (Fig. 4H; Supplementary Figs S3, S4).

With the dihydroflavone eriodictyol as substrate, a 3'-methylation product (homoeriodictyol) and a 4'-methylation product (hesperetin) were both identified; furthermore, the enzyme was also capable of methylating its corresponding glycosides eriocitrin and neoeriocitrin (eriodictyol-7-*O*-rutinoside and eriodictyol-7-*O*-neohesperidoside) (Fig. 4I–K). Methylation of naringenin (which lacks a hydroxyl group in the 3' position compared with eriodictyol) and hesperetin (4'-methylated eriodictyol) by the recombinant enzyme was not observed (Table 1; Supplementary Fig. S5). Caffeic acid also acted as a substrate, and two peaks were detected, which were identified as ferulic acid (its 3-methyl ether) and isoferulic acid (its 4-methyl ether) (Fig. 4L).

### Substrate preference of recombinant CrOMT1

**Fig. 5.**
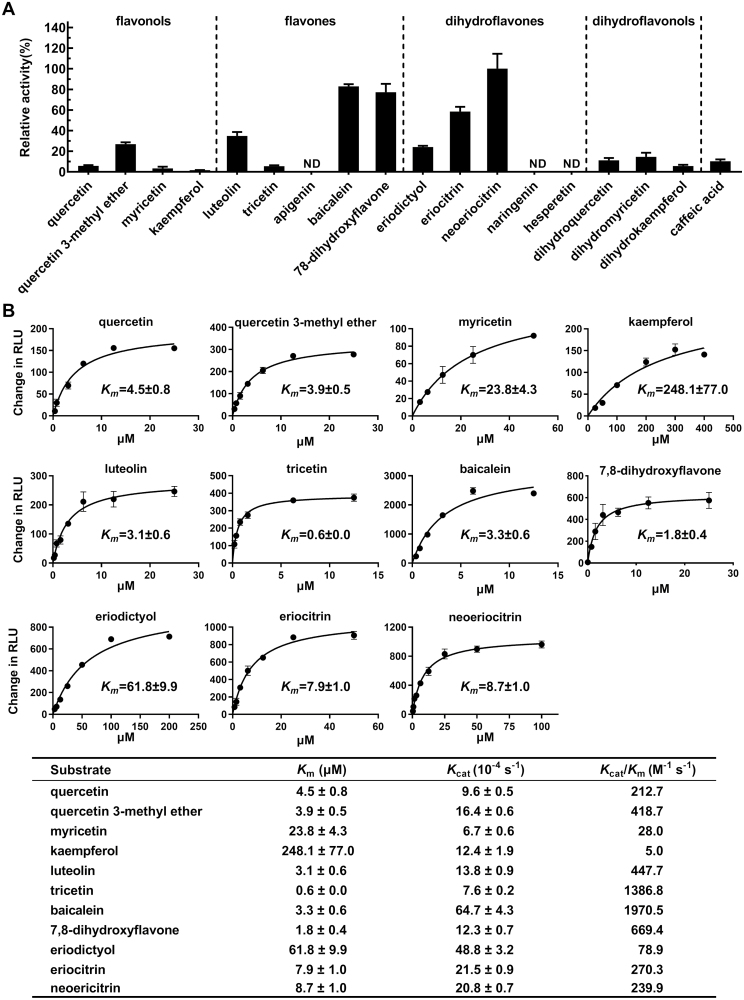
Relative activity and kinetic properties of CrOMT1. (A) Relative activity of CrOMT1 with four groups of flavonoids: dihydroflavonols, dihydroflavones, flavones, and flavonols. ND, not detected. (B) Kinetic properties of CrOMT1 with different phenolic substrates. Kinetic parameters were estimated by non-linear curve fitting using Michaelis–Menten. Change in RLU (relative light units) indicates the background-subtracted RLU for each sample. Values are means ±SE (*n*=3).

Recombinant CrOMT1 was tested for its optimum pH and temperature using luteolin as a substrate (Supplementary Fig. S2). The highest reaction rate was at pH 8.0 (in Tris–HCl buffer) and 40 °C, and further tests were carried out at pH 8.0 and 37 °C. Overall, recombinant CrOMT1 exhibited a preference for flavones and dihydroflavones compared with flavonols and dihydroflavonols (Fig. 5A). In order to investigate the phenolic substrate preference of recombinant CrOMT1, kinetic properties were determined in Tris–HCl at pH 8.0 and 37 °C for 30 min and *K*_m_ and *K*_cat_ values were calculated based on non-linear regression curve fitting using Michaelis–Menten (Fig. 5B). *K*_cat_/*K*_m_ of these substrates tested was also calculated to better evaluate the preferred substrates of CrOMT1, and apparent differences in catalytic efficiencies were observed (Fig. 5B). Specifically, CrOMT1 exhibited the best affinity as well as the highest catalytic efficiency for flavones compared with flavonols and dihydroflavones. The *K*_cat_/*K*_m_ values of the four flavones, baicalein, tricetin, 7,8-dihydroxyflavone, and luteolin, were 1970.5, 1386.8, 669.4, and 447.7 M^–1^ s^–1^, respectively. The catalytic efficiencies of CrOMT1 for quercetin, myricetin, and kaempferol were obviously reduced compared with that of flavones, and the *K*_cat_/*K*_m_ values of the three flavonols were 212.7, 28.0, and 5.0 M^–1^ s^–1^, respectively. Kaempferol, which has no vicinal hydroxy groups, showed the worst affinity (*K*_m_=248.1 μM) toward CrOMT1.The very similar structure of quercetin 3-methyl ether to that of luteolin might account for the similar catalytic efficiency of the two substrates. The catalytic efficiencies for the two glycosides of eriodictyol were more than three times higher than that of eriodictyol due to the much better affinity of the glycosides than the aglycone. Taken together, flavones with vicinal hydroxy moieties were the most favored substrates for CrOMT1, with the methylation sites towards baicalein, 7,8-dihydroxyflavone, and luteolin at the 6, 8, and 3' position, respectively, and toward tricetin mostly at the 3' and 5' position (Figs 4E–H ,5).

### Transient overexpression of *CrOMT1* enhanced PMF accumulation in ‘Ougan’ fruit peel

**Fig. 6.**
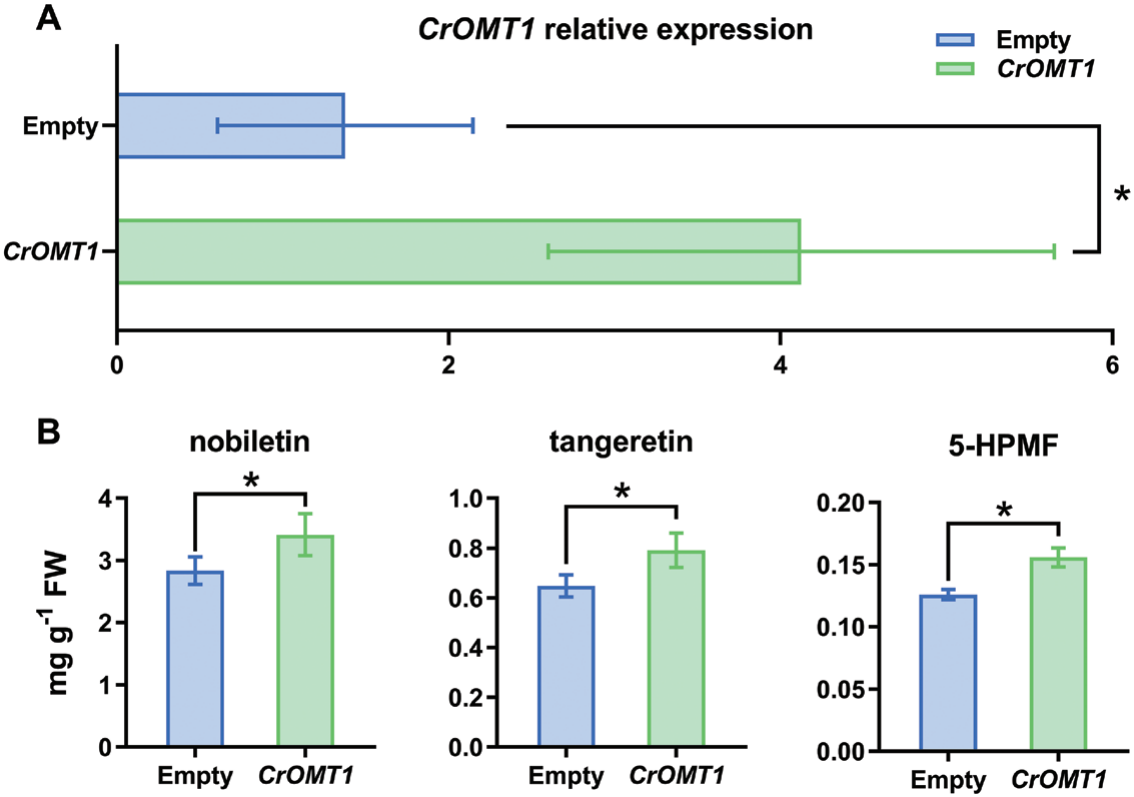
Transient overexpression of *CrOMT1* increases PMF content in ‘Ougan’ fruit peel. (A) Expression profiles of *CrOMT1* after infiltration with *CrOMT1*-pBI121. (B) Changes of PMFs after transient overexpression of *CrOMT1* in ‘Ougan’ fruit peel. The effect of transient expression of *CrOMT1* on the content of the three most abundant PMFs in the peel of ‘Ougan’ fruit was measured after infiltration with *CrOMT1*-pBI121, with empty vector as control: 5-HPMF, 5-hydroxy-6,7,8,3',4'-pentamethoxyflavone. The content of flavonoids is expressed as mg g^–1^ FW. Bars indicated in light blue and light green are empty vector and *CrOMT1*-pBI121, respectively. Values are means ±SE (*n*=7). *P<0.05 (right-tailed paired *t*-test, *n*=7).

‘Ougan’ fruit peel is known to accumulate abundant PMFs (Wang *et al.*, 2017), and transient overexpression experiments were conducted in order to explore the potential role of *CrOMT1* in the methylation of flavonoids *in vivo*. Both the transcript levels of *CrOMT1* and the content of PMFs in ‘Ougan’ peel infiltrated with *CrOMT1*-pBI121 increased significantly compared with that with empty vector (Fig. 6).

## Discussion

In this study, we identified CrOMT1 from citrus as a CCoAOMT-like enzyme that favored flavones with vicinal hydroxy groups *in vitro*. The accumulation profiles of *CrOMT1* transcripts were highly correlated with PMF accumulation during ‘Ougan’ fruit maturation and in response to UV-B irradiation, and transient overexpression of *CrOMT1* in the peel of ‘Ougan’ fruit enhanced the accumulation of PMFs.

### CrOMT1 is a multifunctional enzyme with a preference for flavones

To test the substrate preferences of CrOMT1, a total of four representative classes of flavonoids and a simple phenolic acid (caffeic acid) were tested in *in vitro* assays. As found with other functionally characterized PFOMTs (Ibdah *et al.*, 2003; Widiez *et al.*, 2011; Berim and Gang, 2013; Wils *et al.*, 2013), CrOMT1 could efficiently accept a wide range of phenolic acid with vicinal hydroxy groups. Retention of such a promiscuous catalytic function has been suggested to be an evolutionary advantage (Aharoni *et al.*, 2005; Wils *et al.*, 2013).

Relative activities of various substrates were determined and, among the tested substrates, flavone and dihydroflavones appeared to be favored by CrOMT1 (Fig. 5A). CrOMT1 showed the highest catalytic efficiency towards flavones based on subsequent kinetic analysis and preferred methylation at 6-OH and 8-OH on the A-ring to 3'-OH (Fig. 5B). Similarly, flavones were also favored by ObPFOMT-1 from *O. basilicum*, and methylation at 8-OH was preferred to 3'-OH (Berim and Gang, 2013). ObPFOMT-1, with 60% identity to CrOMT1, has been proposed to be a potential enzyme involved in the biosynthesis of nevadensin, a major methoxylated flavone in some basil cultivars (Berim and Gang, 2013). The dihydroflavone eriodictyol was converted into its 3'- and 4'-methyl ether at a constant ratio of ~20:80 (Fig. 4I), and this methylation pattern towards eriodictyol was identical to that of AtCCoAOMT7 from *A. thaliana* (Wils *et al.*, 2013). When eriocitrin and neoeriocitrin, two glycosides of eriodictyol ubiquitous in citrus, were tested, CrOMT1 had significantly increased affinity for these substrates (Fig. 5B). Several CCoAOMT-like enzymes involved in methylated flavonoids also displayed much better activity with glycosides compared with the corresponding aglycone (Ibdah *et al.*, 2003; Hugueney *et al.*, 2009; Gomez Roldan *et al.*, 2014; Du *et al.*, 2015). CrOMT1 had lower catalytic activity towards flavonols. The methylation pattern for quercetin and myricetin was different from that of most PFOMTs, which usually displayed methylation at the *meta*-position. Notably, the sequential methylation at the 3' and 4' position toward quercetin is the first report to our knowledge, and the unique capability could be used in enzyme engineering for generating quercetin 3',4'-dimethyl ether.

### CrOMT1 is a novel CCoAOMT-like enzyme from citrus

The existence of OMTs with different methylation patterns towards flavonoids has been revealed in the cell-free extracts of citrus (Brunet *et al.*, 1978; Brunet and Ibrahim, 1980), and in this report we identified CrOMT1 from ‘Ougan’ fruit as playing a role in flavonoid *O*-methylation. CrOMT1 has 96.8% identity to Cs1g22450 from sweet orange, a putative OMT (Supplementary Table S3); however, following a previous systematic analysis of OMTs in citrus, the latter enzyme was not suggested to be involved in flavonoid *O*-methylation because the selection criteria were restricted to members of the COMT subfamily (Liu *et al.*, 2016). CdFOMT5 from *Citrus depressa* was identified as a COMT-type flavonoid-*O*-methyltransferase (Itoh *et al.*, 2016), with 97.2% identity to Ciclev10031952m, a putative OMT in our study. Recombinant CdFOMT5 preferred flavonols to flavones and methylated flavones at the 3, 5, 6, and 7 position *in vitro*, although the 6-methylation was negligible. CrOMT1 has distinct catalytic properties compared with CdFOMT5, and the recombinant CrOMT1 favored flavones and exhibited the highest catalytic efficiency with the 6-, 8-, and 3'-OH groups of PMF-type flavones. Phylogenetic analysis (Fig. 3) and functional characterization *in vitro* showed that CrOMT1 was a new member of the PFOMTs or CCoAOMT-like subgroup (Ibdah *et al.*, 2003) that could be involved in the methylation of flavonoids in citrus.

### *CrOMT1* is responsible for PMF biosynthesis in ‘Ougan’ fruit peel

Three major PMFs (nobiletin, tangeretin, and 5-HPMF) exist in ‘Ougan’ fruit, and they all have three methoxy groups on the A-ring (6,7,8-OCH_3_) and one methoxy group on B-ring (4'-OCH_3_) (Fig. 1C). The similar structure of the three PMFs indicated that they might be derived from common precursors *in vivo*, with their structure as 5,6,7,8,3',4'-hexahydroxy flavones except for tangeretin which has no substitution at the 3' position. Although the natural PMF-type substrates were not available for *in vitro* studies, several substrate analogs, including baicalein (6,7,8-trihydroxyflavone), 7,8-dihydroxyflavone, and luteolin (5,7,3',4'-tetrahydroxyflavone), were demonstrated to be favored by recombinant CrOMT1. In addition to the three most abundant PMFs in citrus, several methylated flavonoids were also detected in a trace amount at the mass spectrometric level, such as tricin (Wang *et al.*, 2016). Tricetin was converted into four products by CrOMT1 *in vitro*, and one of them was tricin (Fig. 4F). CrOMT1 showed the best affinity (*K*_*m*_=0.6 μM) and high catalytic efficiency (*K*_cat_/*K*_m_=1386.8 M^–1^ s^–1^) toward tricetin (Fig. 5); therefore, CrOMT1 might also participate in the biosynthesis of the low-accumulated tricin *in vivo*.

Analysis of PMF contents in ‘Ougan’ fruit peel indicated a dramatic tissue-specific accumulation pattern for three major PMFs, which were mainly detected in the flavedo of ‘Ougan’ fruit (Fig. 1A). These results indicated that *CrOMT1* was highly correlated with PMF accumulation by both tissue and developmental stages of ‘Ougan’ fruit. Furthermore, transcript abundance of *CrOMT1* sharply increased after UV-B irradiation, and the accumulation of three PMFs also increased (Figs 1B, 2A). Phenolic compounds, especially flavonoids, are thought to serve as a UV-absorbing sunscreen in epidermal tissues of plants (Li *et al.*, 1993; Landry *et al.*, 1995; Rozema *et al.*, 1997; Agati and Tattini, 2010), and several upstream genes involved in the flavonoid pathway are also induced by UV-B irradiation (Falcone Ferreyra *et al.*, 2010; Huang *et al.*, 2016; Henry-Kirk *et al.*, 2018). Similarly, it has been shown that methylation of flavonoids was induced by high light irradiation in the ice plant (*M. crystallinum*), and the transcript level of *McPFOMT* rapidly increased after exposure to elevated light conditions (Ibdah et al., 2002, 2003). Notably, McPFOMT along with CCoAOMT7 from *A. thaliana* and CCoAOMT from chickweed (*Stellaria longipes*) were proposed to form the new subclass (PFOMTs subgroup) within the CCoAOMT subfamily, with potential function not restricted to lignin biosynthesis (Ibdah *et al.*, 2003).

Transient overexpression of *CrOMT1* in the peel of ‘Ougan’ fruit enhanced the accumulation of the three most abundant PMFs (Fig. 6). Kinetic analyses (Fig. 5B) confirmed that recombinant CrOMT1 preferred to accept flavones with vicinal hydroxyl groups *in vitro*, which are similar to potential PMF-type substrates. This substrate preference is consistent with the accumulation of PMFs in response to overexpression of *CrOMT1*.In summary, we characterized a CCoAOMT-like enzyme, CrOMT1, in citrus for the first time. After testing a wide range of substrates *in vitro*, we concluded that CrOMT1 showed a strong preference for flavones, especially those with methylation at the 6 and 8 positions, which were vital sites for PMFs synthesis. This together with the high correlation between accumulation of *CrOMT1* transcripts and PMF accumulation both developmentally and in response to UV-B irradiation and the enhanced PMF accumulation in the peel of ‘Ougan’ overexpressing *CrOMT1* support the suggestion that CrOMT1 is responsible for PMFs biosynthesis in ‘Ougan’ fruit peel. Identification of multifunctional enzymes responsible for production of methylated products is important for the synthesis of various methylated flavonoids by enzyme engineering and for bioactivity research.

## Supplementary data

Supplementary data are available at *JXB* online.

Fig. S1. Sequence alignment of CrOMT1 with other members of the CCoAOMT subfamily.

Fig. S2. Characterization of recombinant CrOMT1.

Fig. S3. MS/MS spectrometry of methylated products generated by CrOMT1 *in vitro* and corresponding authentic standards.

Fig. S4. ^1^H and ^13^C NMR data of 7,8-dihydroxyflavone and its methylated product by CrOMT1.

Fig. S5. Overview of the methylation of substrates by CrOMT1.

Fig. S6. HPLC chromatograms of time-dependent methylation of quercetin by recombinant CrOMT1 *in vitro*.

Table S1. Primers used for cloning *CrOMT1* and qPCR.

Table S2. UniProt entries of proteins from other organisms used in phylogenetic analysis.

Table S3. Fifty-five *OMT* genes detected in the flavedo of ‘Ougan’ fruit.

Table S4. Percentage amino acid identity shared by PFOMTs described in the phylogenetic tree.

Table S5. Accumulation of the three most abundant PMFs in two different tissues during ‘Ougan’ fruit maturation.

eraa083_suppl_Supplementary_Figures_S1-S6_Tables_S1-S5
