# Supplementary material for: Characterization of a caffeoyl-CoA O-methyltransferase-like enzyme involved in biosynthesis of polymethoxylated flavones in Citrus reticulata
Source: J Exp Bot. 2020 Mar 17;71(10):3066–79. doi: 10.1093/jxb/eraa083 (PMC7475179; doi:10.1093/jxb/eraa083)
Supplement: eraa083_suppl_Supplementary_Figures_S1-S6_Tables_S1-S5 [file eraa083_suppl_supplementary_figures_s1-s6_tables_s1-s5.pdf]

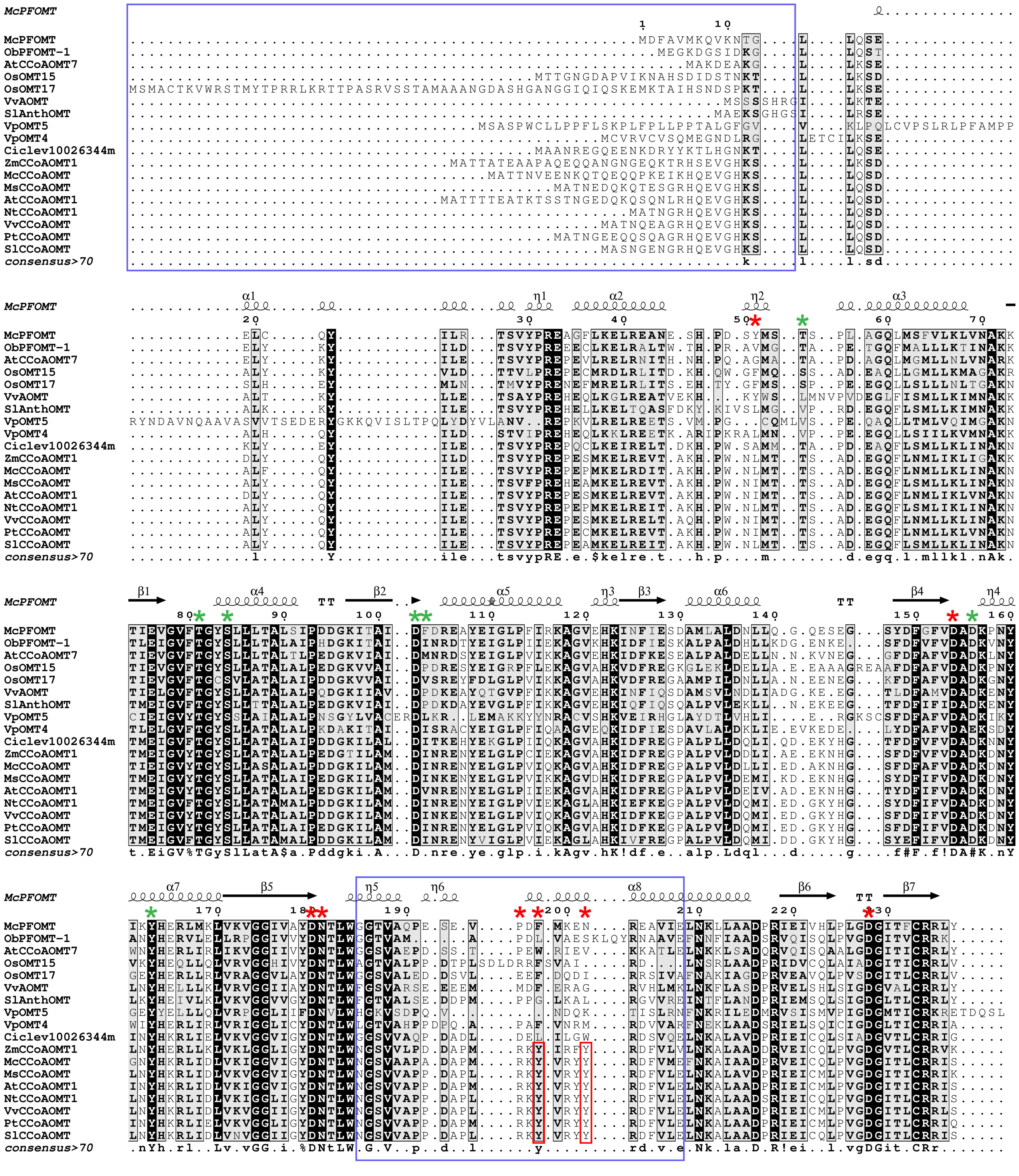

Fig. S1 Sequence alignment of CrOMT1 with other members of the CCoAOMT subfamily.

CrOMT1 in this paper was shown as its accession number (Ciclev10026344m) in *Citrus clementina* genome. At the top of sequences block, secondary structure of McPFOMT was displayed. And numbering

of McPFOMT protein is dotted every 10th position. Helices and β-strands are depicted as coils and arrows, respectively, and are numbered. α, β, η and T indicate α-helix, β-strand, 3/10-helix and turn, respectively. Below the aligned sequences, a consensus sequence with consensus level over 0.7 is shown. Fully conserved residues are shown in white characters boxed in black shading. Similar residues are shown in black bold characters and boxed with gray shading. Potential residues important for catalysis, substrate binding or substrate specificity are indicated with asterisks above the sequence block according to Walker *et al.* (2016). Residues involved in SAM binding are depicted as green asterisks. Residues associated with Caffeoyl-CoA binding are boxed in red. Two regions (N-terminus and a variable insertion loop near C-terminus) important for substrate specificity are boxed in blue (Kopycki *et al.*, 2008).

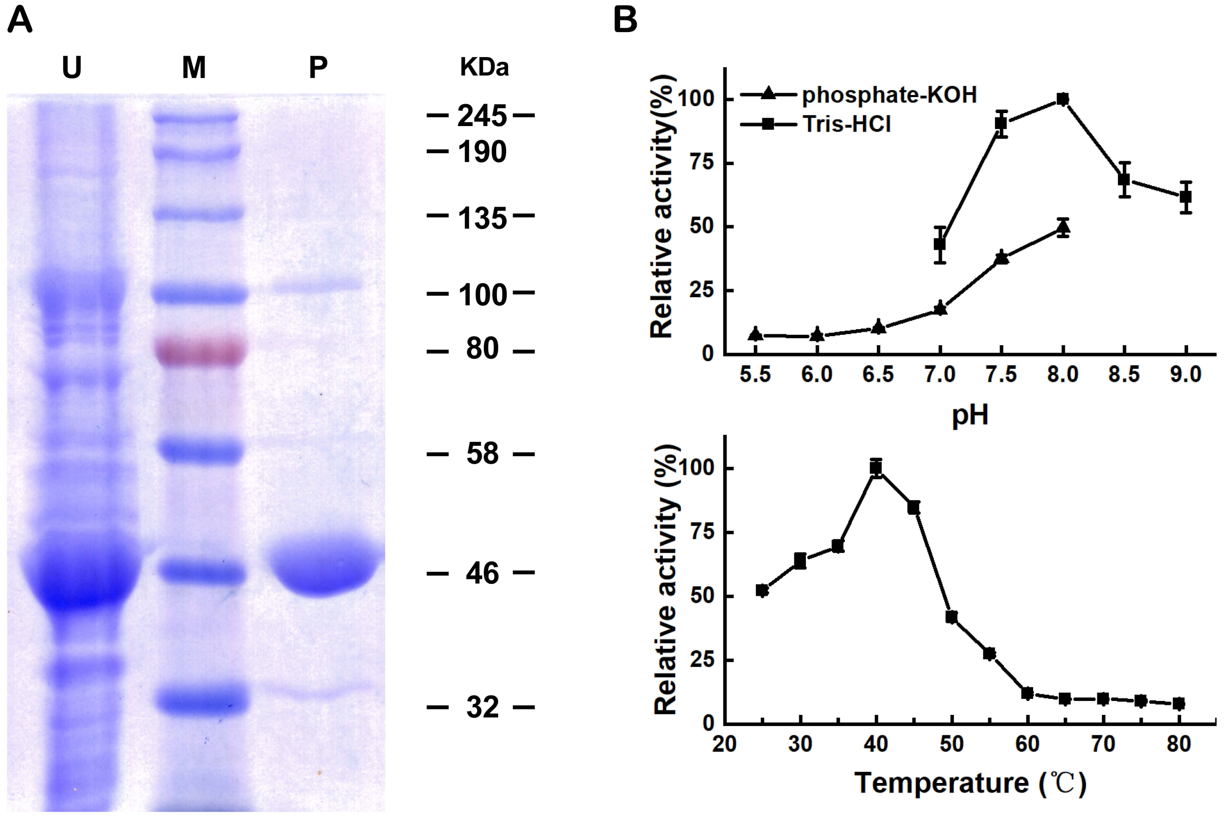

Fig. S2 Characterization of recombinant CrOMT1.

(A) SDS–PAGE analysis of the recombinant enzyme expressed in *E. coli*.

U, unpurified fractions after induction with 1 mM IPTG; M, protein molecular weight standards; P, purified enzyme.

(B) The effect of pH and temperature on the enzyme activity.

Assays were performed with luteolin as a substrate, and the peak area of its methylated product chrysoeriol was used to determine the relative activity of the enzyme. Values are means ±SE (*n*=3).

quercetin methylated product 1

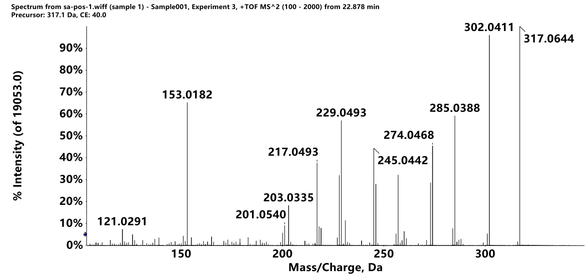

isorhamnetin standard

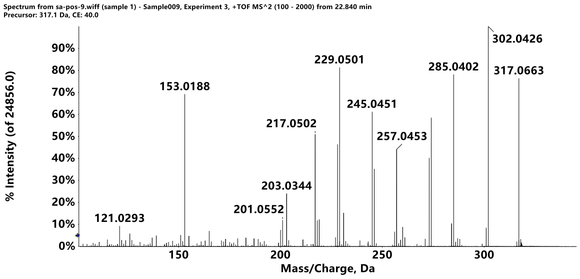

quercetin methylated product 2

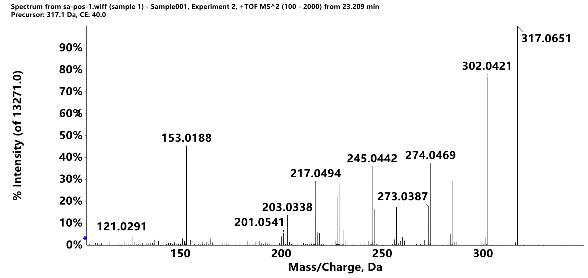

tamarixetin standard

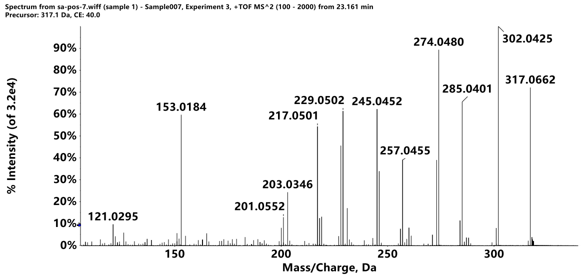

quercetin methylated product 3

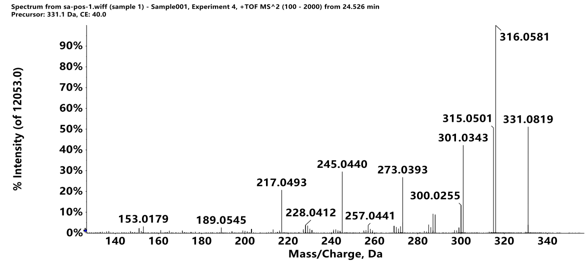

quercetin 3-methyl ether methylated product

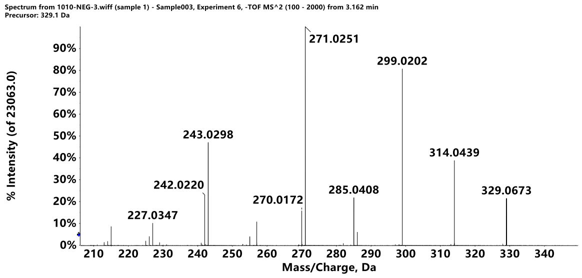

quercetin 3,3'-dimethyl ether standard

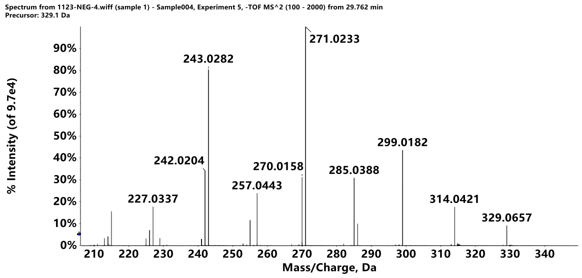

myricetin methylated product 1

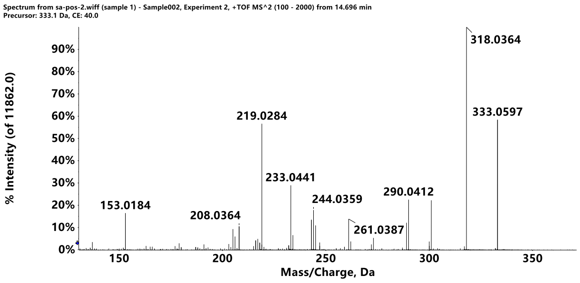

laricitrin standard

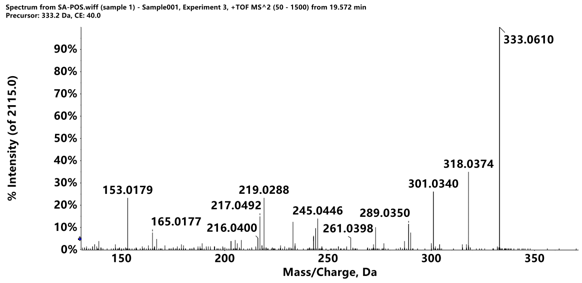

myricetin methylated product 2

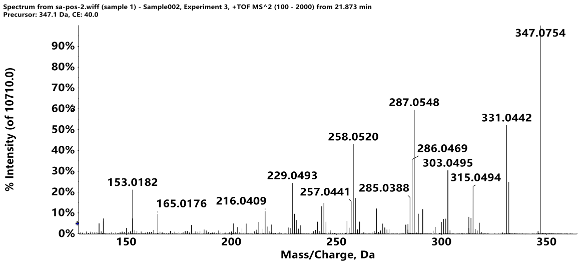

syringetin standard

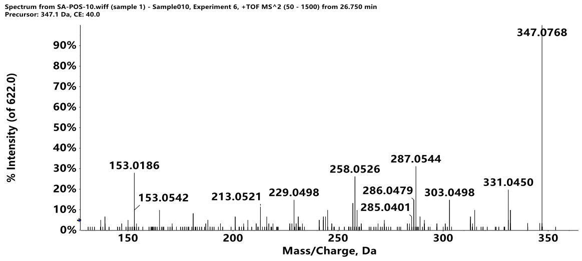

myricetin methylated product 3

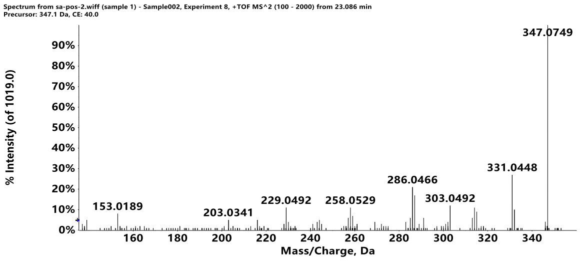

kaempferol methylated product

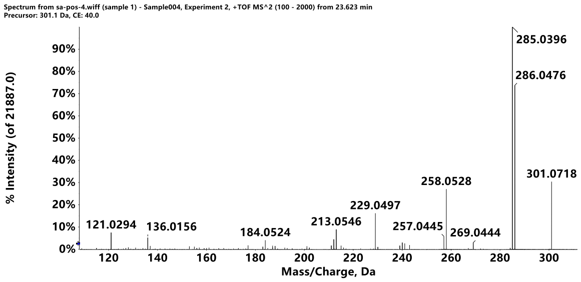

isokaempferide standard

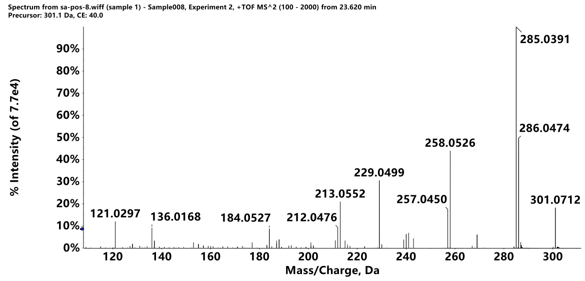

luteolin methylated product

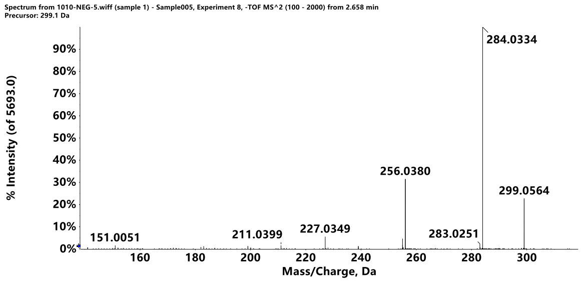

chrysoeriol standard

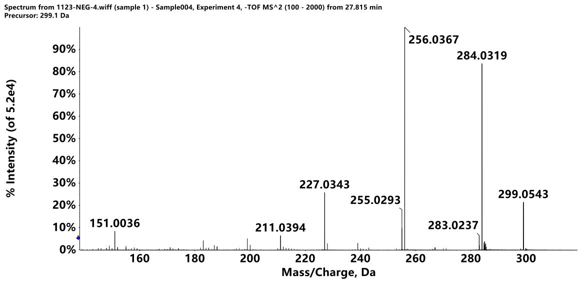

tricetin methylated product 1

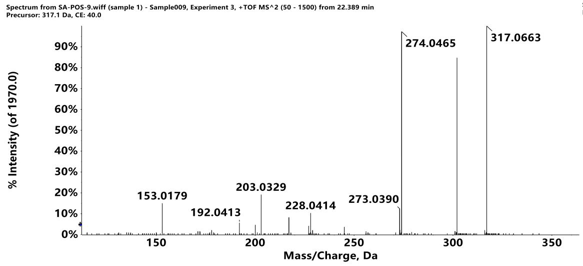

tricetin methylated product 2

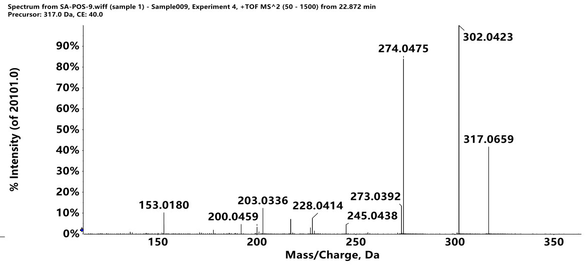

tricetin methylated product 3

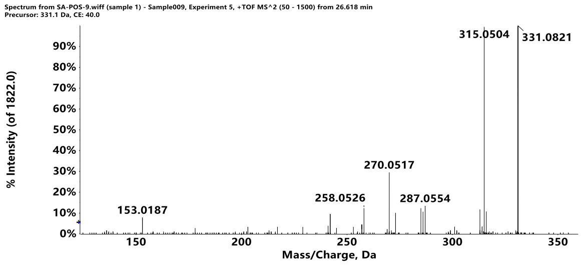

tricetin methylated product 4

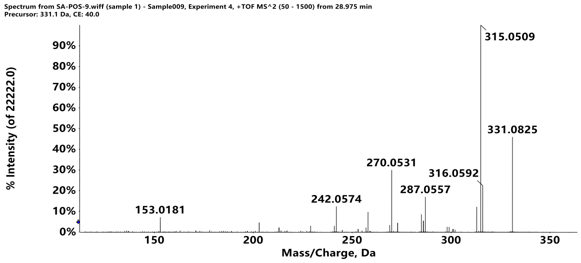

tricin standard

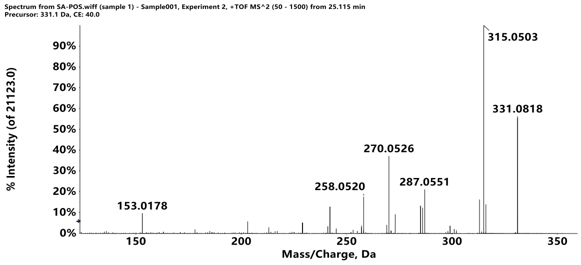

baicalein methylated product

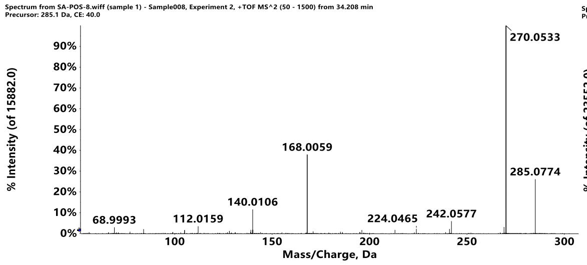

oroxylin A standard

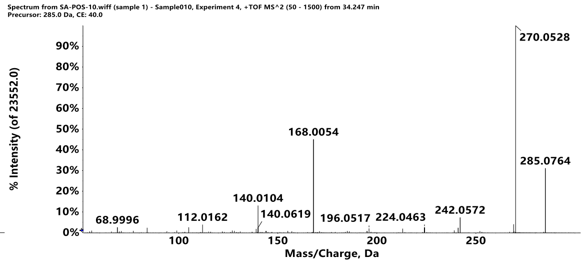

7,8-dihydroxyflavone methylated product

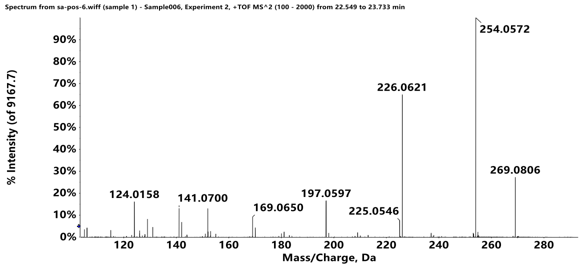

eriodictyol methylated product 1

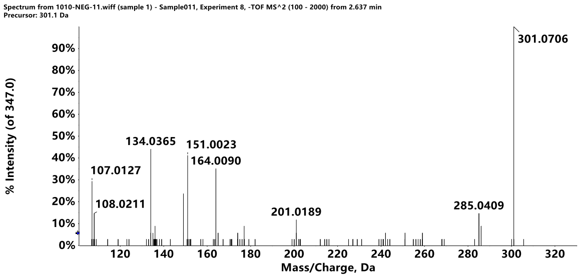

homoeriodictyol standard

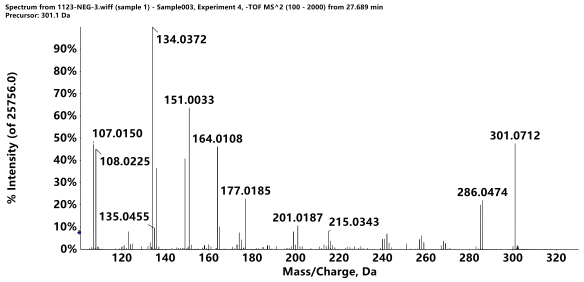

eriodictyol methylated product 2

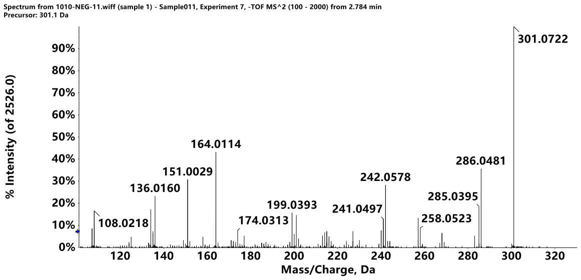

hesperetin standard

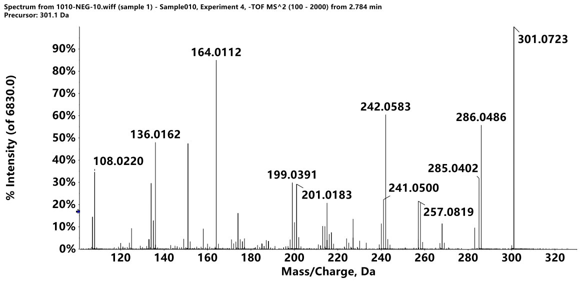

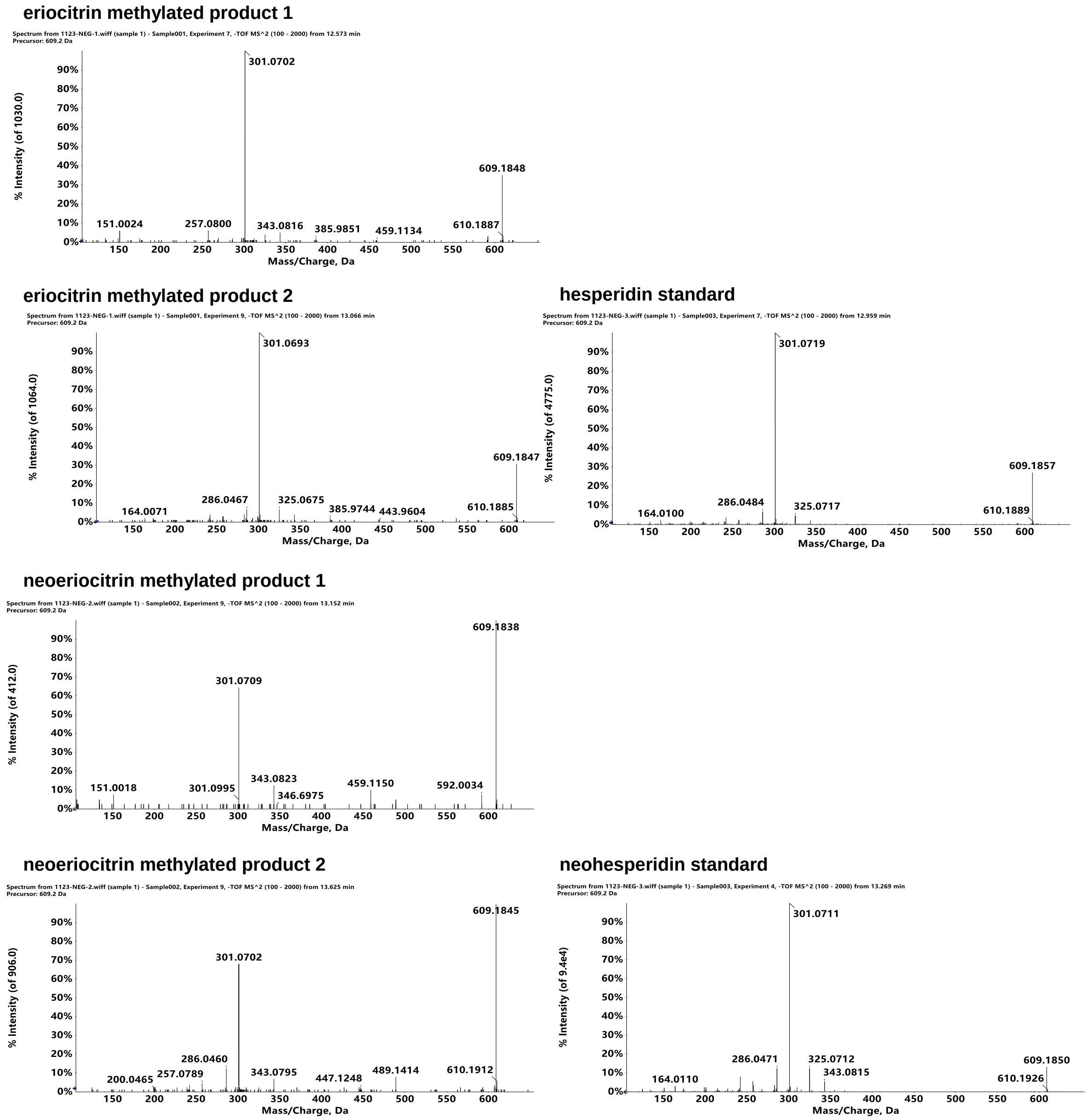

Fig. S3 MS/MS spectrometry of methylated products generated by CrOMT1 *in vitro* and corresponding authentic standards.

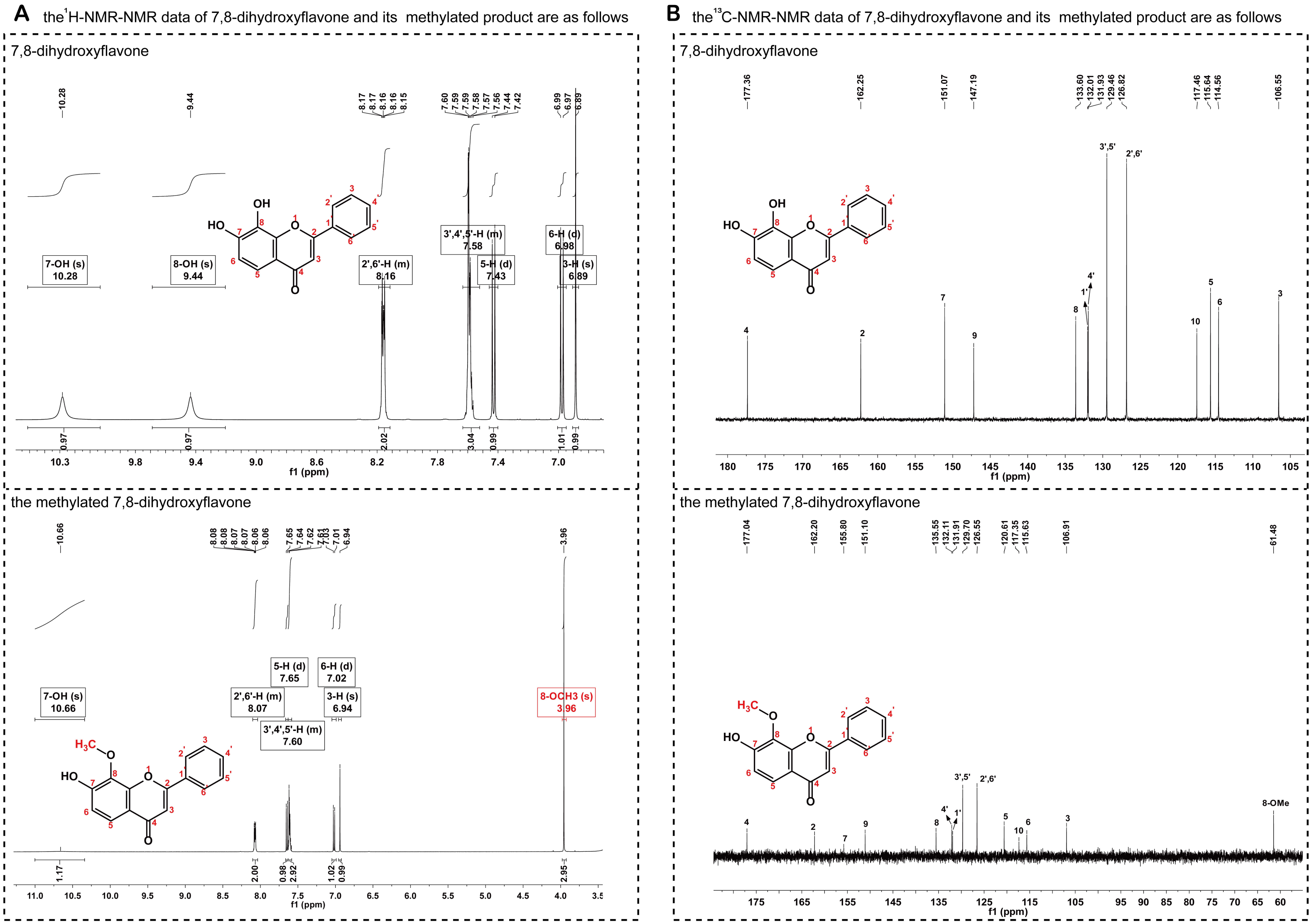

Measured in DMSO-d6 at 500 MHZ

Measured in DMSO-d6 at 126 MHZ

Fig. S4 ^1H and ^{13}C NMR data of 7,8-dihydroxyflavone and its methylated product by CrOMT1.

(A) the ^1H-NMR data of substrate and product are as follows:

7,8-dihydroxyflavone: ^1H NMR (500 MHz, DMSO) δ 10.28 (s, 1H), 9.44 (s, 1H), 8.19 – 8.11 (m, 2H), 7.63 – 7.52 (m, 3H), 7.43 (d, J = 8.6 Hz, 1H), 6.98 (d, J = 8.6 Hz, 1H), 6.89 (s, 1H).

The methylated 7,8-dihydroxyflavone: ^1H NMR (500 MHz, DMSO) δ 10.66 (s, 1H), 8.10 – 8.03 (m, 2H), 7.65 (d, J = 8.8 Hz, 1H), 7.63 – 7.58 (m, 3H), 7.02 (d, J = 8.8 Hz, 1H), 6.94 (s, 1H), 3.96 (s, 3H).

(B) the ^{13}C-NMR data of substrate and product are as follows:

7,8-dihydroxyflavone: ^{13}C NMR (126 MHz, DMSO) δ 177.36, 162.25, 151.07, 147.19, 133.60, 132.01, 131.93, 129.46, 126.82, 117.46, 115.64, 114.56, 106.55.

The methylated 7,8-dihydroxyflavone: ^{13}C NMR (126 MHz, DMSO) δ 177.04, 162.20, 155.80, 151.10, 135.55, 132.11, 131.91, 129.70, 126.55, 120.61, 117.35, 115.63, 106.91, 61.48.

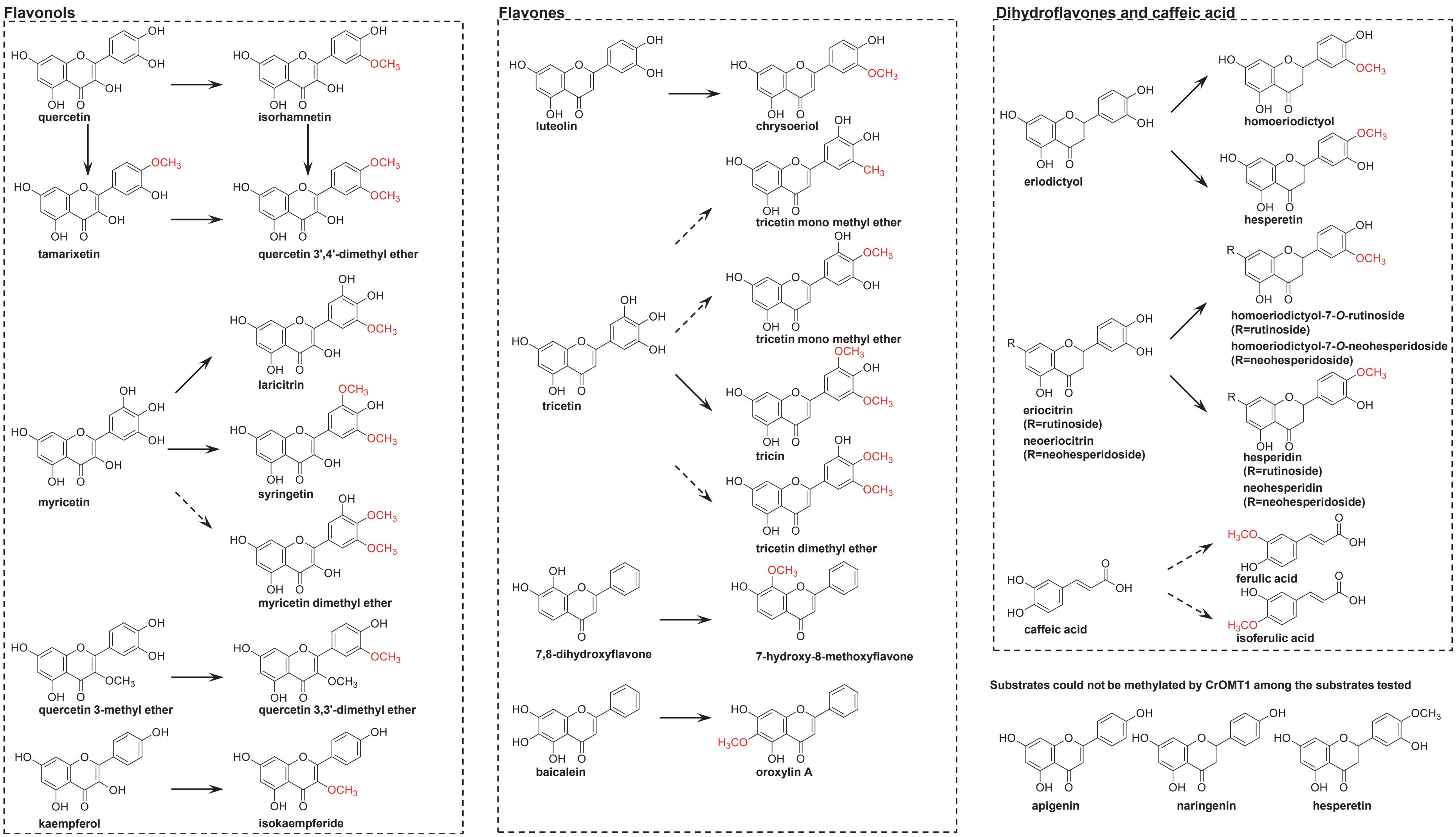

Fig. S5 Overview of the methylation of substrates by CrOMT1.

Trivial names and structural formule of flavonols, flavones, dihydroflavones and caffeic acid are shown, together with their methylated

products generated by CrOMT1. The methoxy groups transferred by the enzyme are indicated in red. Solid arrow represents the reaction products verified by retention time and MS/MS data in comparison with authentic standards or NMR spectroscopy. Dashed arrows represent the probable reaction product inferred by retention time, MS/MS data or literature. Substrates that could not be converted are also shown.

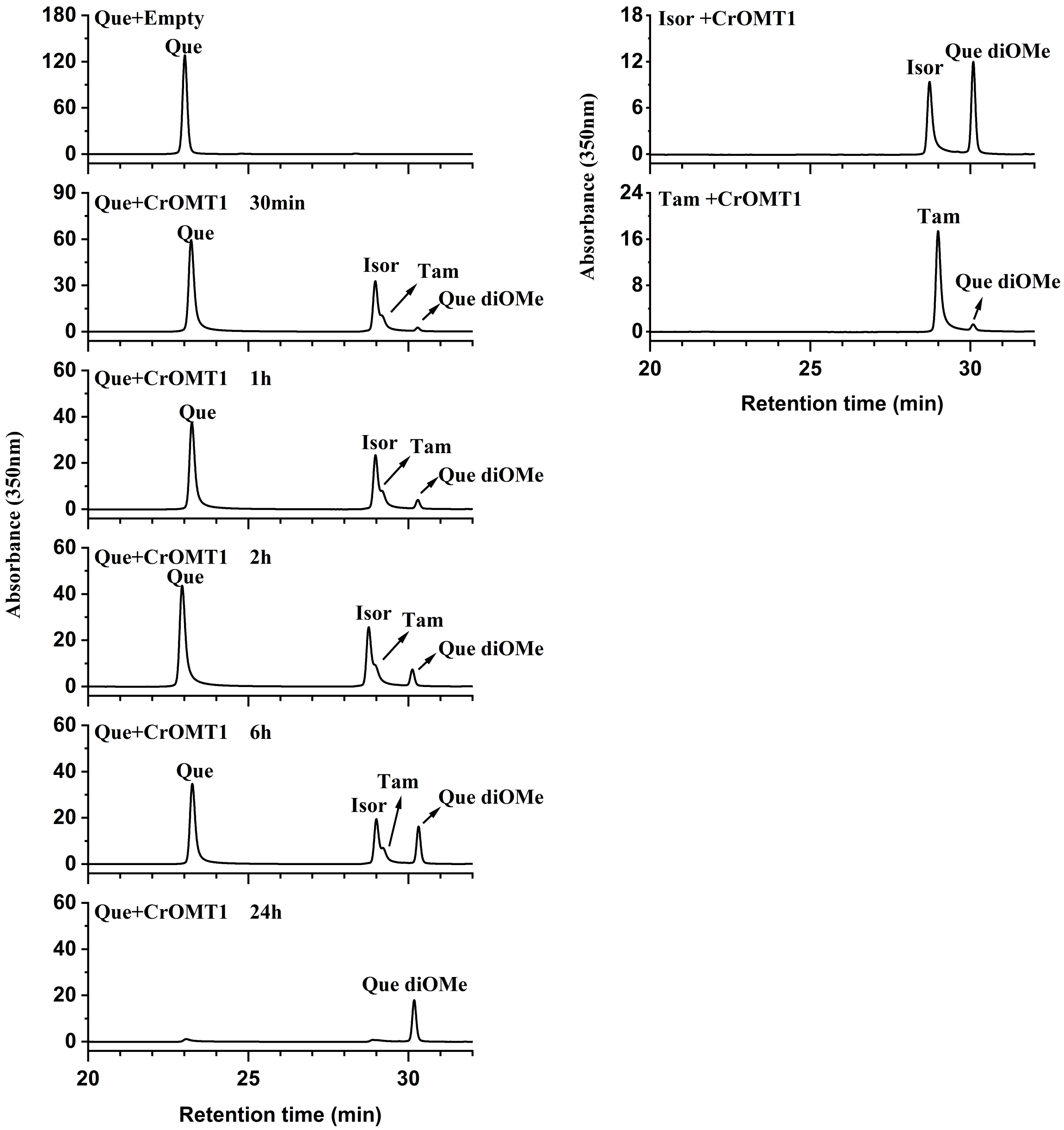

Fig. S6 HPLC chromatograms of time-dependent methylation of quercetin by recombinant CrOMT1 *in vitro*.

(A) HPLC chromatograms of quercetin methylation when incubated with recombinant CrOMT1 for 30 min, 1 h, 2 h, 6 h and 24 h, respectively.

(B) HPLC chromatograms of isorhamnetin and tamarixetin methylated by CrOMT1.

Que, quercetin; Isor, isorhamnetin; Tam, tamarixetin; Que diOMe, quercetin dimethyl ether.

Table S1. Primers used for cloning *CrOMT1* and qPCR.

Gene	Forward primer (5' to 3')	Reverse primer (5' to 3')
CrOMT1 -CDs	ATATGGCTGCGAATCGGGAAG	TCAGCCAATGCGACGACA
CrOMT1 -PET	TCAGGATCCATGGCTGCGAATCGGGAAG	CGACTCGAGGCCAATGCGACGACATAATG
CrOMT1 -pBI121	GGACTCTAGAGGATCCATGGCTGCGAATCGGGAAG	GACCACCCGGGGATCCGCCAATGCGACGACATAATG
CrOMT1- qPCR	ATGCCGACCTGGATGAGCTA	GAGAGGATCAGCCAATGCGA
CrOMT1- qPCR(for transient overexpression)	GAGCTAATCTTAGGCTGGAGGG	GCGACGACATAATGTAACCCC
Ciclev10005275m- qPCR	GTGTATGATCAATCAGGCAAGC	CAGAAAGAGTGATGGCCCGT
Ciclev10005276m- qPCR	AAGCTATTCTCCGACGCTGG	AAGAGCAGATGCTGGAAACAA
Ciclev10005279m- qPCR	GTGTGTGATCAATCAGGCAAG	GTGCCGAGACCAACTCAGAA
Ciclev10012033m- qPCR	TTACTCACCGGAACAGAGCG	GGTCAAATCGATGGAGAATCCAAG
Ciclev10015723m- qPCR	CGTGGAAGTGAGAGGACTGAG	CAAACATGAGTACAAGCGCAA
Ciclev10020814m- qPCR	GTGGTGGAATTGGAGCCACA	TCAAGAATTTCACGCAGTGCT
Ciclev10029158m- qPCR	CAATTCTCTGAGGGGGTCCG	TCTCGACAGTTTAACAGTGCTTT
Ciclev10031951m- qPCR	TGAAATAAATGGGCGCTCAACA	GCCAATTGGAAACACGCAATCT
Citrus β-actin-qPCR	CATCCCTCAGCACCTTCC	CCAACCTTAGCACTTCTCC

Underlined sequences represent restriction enzyme site

Table S2. UniProt entries of proteins from other organisms used in phylogenetic analysis.

UniProt entry	Protein name	Organism	Protein family	OMTs subgruop
Q9C5D7	AtCCoAOMT7	*Arabidopsis thaliana* (Mouse-ear cress)	CCoAMT subfamily	PFOMTs
Q6YI95	McPFOMT	*Mesembryanthemum crystallinum* (Common ice plant) (*Cryophytum crystallinum*)	CCoAMT subfamily	PFOMTs
S5DQS6	ObPFOMT-1	*Ocimum basilicum* (Sweet basil)	CCoAMT subfamily	PFOMTs
Q7F8T6	OsOMT17	*Oryza sativa* subsp. *japonica* (Rice)	CCoAMT subfamily	PFOMTs
Q9XGP7	OsOMT15	*Oryza sativa* subsp. *japonica* (Rice)	CCoAMT subfamily	PFOMTs
K4CVP2	SlAnthOMT	*Solanum lycopersicum* (Tomato) (*Lycopersicon esculentum*)	CCoAMT subfamily	PFOMTs
F2YP46	VpOMT5	*Vanilla planifolia* (Vanilla)	CCoAMT subfamily	PFOMTs
F2YP45	VpOMT4	*Vanilla planifolia* (Vanilla)	CCoAMT subfamily	PFOMTs
C7AE94	VvAOMT	*Vitis vinifera* (Grape)	CCoAMT subfamily	PFOMTs
O49499	AtCCoAOMT1	*Arabidopsis thaliana* (Mouse-ear cress)	CCoAMT subfamily	true CCoAOMTs
Q40313	MsCCoAOMT	*Medicago sativa* (Alfalfa)	CCoAMT subfamily	true CCoAOMTs
O65162	McCCoAOMT	*Mesembryanthemum crystallinum* (Common ice plant) (*Cryophytum crystallinum*)	CCoAMT subfamily	true CCoAOMTs
O24144	NtCCoAOMT1	*Nicotiana tabacum* (Common tobacco)	CCoAMT subfamily	true CCoAOMTs
O24149	NtCCoAOMT2	*Nicotiana tabacum* (Common tobacco)	CCoAMT subfamily	true CCoAOMTs
Q42945	NtCCoAOMT6	*Nicotiana tabacum* (Common tobacco)	CCoAMT subfamily	true CCoAOMTs
O24151	NtCCoAOMT4	*Nicotiana tabacum* (Common tobacco)	CCoAMT subfamily	true CCoAOMTs
O04899	NtCCoAOMT5	*Nicotiana tabacum* (Common tobacco)	CCoAMT subfamily	true CCoAOMTs
O24150	NtCCoAOMT3	*Nicotiana tabacum* (Common tobacco)	CCoAMT subfamily	true CCoAOMTs
Q43095	PtCAMT	*Populus tremuloides* (Quaking aspen)	CCoAMT subfamily	true CCoAOMTs
O65862	PtCCoAOMT1	*Populus trichocarpa* (Western balsam poplar) (*Populus balsamifera* subsp. *trichocarpa*)	CCoAMT subfamily	true CCoAOMTs
O65922	PtCCoAOMT2	*Populus trichocarpa* (Western balsam poplar) (*Populus balsamifera* subsp. *trichocarpa*)	CCoAMT subfamily	true CCoAOMTs
A8IFQ7	SlCCoAOMT	*Solanum lycopersicum* (Tomato) (*Lycopersicon esculentum*)	CCoAMT subfamily	true CCoAOMTs
Q43237	VvCCoAOMT	*Vitis vinifera* (Grape)	CCoAMT subfamily	true CCoAOMTs
Q9XGD6	ZmCCoAOMT1	*Zea mays* (Maize)	CCoAMT subfamily	true CCoAOMTs
Q9XGD5	ZmCCoAOMT2	*Zea mays* (Maize)	CCoAMT subfamily	true CCoAOMTs
Q9FK25	AtOMT1	*Arabidopsis thaliana* (Mouse-ear cress)	COMT subfamily	COMTs
Q8GSN1	CrOMT2	*Catharanthus roseus* (*Madagascar periwinkle*) (Vinca rosea)	COMT subfamily	COMTs
Q8W013	CrCOMT1	*Catharanthus roseus* (*Madagascar periwinkle*) (Vinca rosea)	COMT subfamily	COMTs
Q6VCW3	CrOMT6	*Catharanthus roseus* (*Madagascar periwinkle*) (Vinca rosea)	COMT subfamily	COMTs
Q42653	CaOMT2	*Chrysosplenium americanum* (Golden saxifrage)	COMT subfamily	COMTs
P59049	CaOMT1	*Chrysosplenium americanum* (Golden saxifrage)	COMT subfamily	COMTs
A0A120MXL9	CdFOMT3	*Citrus depressa*	COMT subfamily	COMTs
A0A125T1T4	CdFOMT1	*Citrus depressa*	COMT subfamily	COMTs
A0A120MXM2	CdFOMT4	*Citrus depressa*	COMT subfamily	COMTs
A0A125T1T5	CdFOMT5	*Citrus depressa*	COMT subfamily	COMTs
A0A120MXM3	CdFOMT6	*Citrus depressa*	COMT subfamily	COMTs
Q43771	HvOMT1	*Hordeum vulgare* (Barley)	COMT subfamily	COMTs
A5YTR4	Hv7OMT	*Hordeum vulgare* subsp. *vulgare* (Domesticated barley)	COMT subfamily	COMTs
P28002	MsCOMT1	*Medicago sativa* (Alfalfa)	COMT subfamily	COMTs
Q6VMW0	MpOMT2	*Mentha piperita* (Peppermint) (*Mentha aquatica* x *Mentha spicata*)	COMT subfamily	COMTs
Q6VMV9	MpOMT3	*Mentha piperita* (Peppermint) (*Mentha aquatica* x *Mentha spicata*)	COMT subfamily	COMTs
Q6VMW2	MpOMT1A	*Mentha piperita* (Peppermint) (*Mentha aquatica* x *Mentha spicata*)	COMT subfamily	COMTs
Q6VMW1	MpOMT1B	*Mentha piperita* (Peppermint) (*Mentha aquatica* x *Mentha spicata*)	COMT subfamily	COMTs
Q6VMV8	MpOMT4	*Mentha piperita* (Peppermint) (*Mentha aquatica* x *Mentha spicata*)	COMT subfamily	COMTs
K0I986	ObFOMT6	*Ocimum basilicum* (Sweet basil)	COMT subfamily	COMTs
K0I977	ObFOMT1	*Ocimum basilicum* (Sweet basil)	COMT subfamily	COMTs
K0I210	ObFOMT4	*Ocimum basilicum* (Sweet basil)	COMT subfamily	COMTs
K0I7Q2	ObFOMT3	*Ocimum basilicum* (Sweet basil)	COMT subfamily	COMTs
K0II72	ObFOMT2	*Ocimum basilicum* (Sweet basil)	COMT subfamily	COMTs
K0ICR0	ObFOMT5	*Ocimum basilicum* (Sweet basil)	COMT subfamily	COMTs
S5DWK8	ObF8OMT-1	*Ocimum basilicum* (Sweet basil)	COMT subfamily	COMTs
Q6ZD89	OsOMT1	*Oryza sativa* subsp. *japonica* (Rice)	COMT subfamily	COMTs
Q0IP69	OsNOMT	*Oryza sativa* subsp. *japonica* (Rice)	COMT subfamily	COMTs
Q43046	PkHOMT1	*Populus kitakamiensis* (Aspen) (*Populus sieboldii* x *Populus grandidentata*)	COMT subfamily	COMTs
Q43047	PkHOMT3	*Populus kitakamiensis* (Aspen) (*Populus sieboldii* x *Populus grandidentata*)	COMT subfamily	COMTs
Q00763	PtOMT1	*Populus tremuloides* (Quaking aspen)	COMT subfamily	COMTs
Q41086	PtOMT2	*Populus tremuloides* (Quaking aspen)	COMT subfamily	COMTs
F2YTN5	ShMOMT2	*Solanum habrochaites* (Wild tomato) (*Lycopersicon hirsutum*)	COMT subfamily	COMTs
F2YTN4	ShMOMT1	*Solanum habrochaites* (Wild tomato) (*Lycopersicon hirsutum*)	COMT subfamily	COMTs
M9Z1G5	ShMOMT3	*Solanum habrochaites* (Wild tomato) (*Lycopersicon hirsutum*)	COMT subfamily	COMTs
A0A088MF62	SlMOMT4	*Solanum lycopersicum* (Tomato) (*Lycopersicon esculentum*)	COMT subfamily	COMTs
A8QW53	SbOMT3	*Sorghum bicolor* (Sorghum) (*Sorghum vulgare*)	COMT subfamily	COMTs
A8QW52	SbOMT1	*Sorghum bicolor* (Sorghum) (*Sorghum vulgare*)	COMT subfamily	COMTs
Q38J50	TaOMT2	*Triticum aestivum* (Wheat)	COMT subfamily	COMTs
Q84N28	TaOMT1	*Triticum aestivum* (Wheat)	COMT subfamily	COMTs
B6VJS4	VvROMT	*Vitis vinifera* (Grape)	COMT subfamily	COMTs
Q6VWG3	ZmOMT1	*Zea mays* (Maize)	COMT subfamily	COMTs

Table S3. Fifty-five *OMT* genes detected in the flavedo of 'Ougan' fruit.

Gene locus name	Gene name	FPKM values of *OMTs* during 'Ougan' fruit maturation				Corelation vs nobiletin (r)	Corelation vs tangeretin (r)	Corelation vs 5-HPMF (r)	Best hit in *C. sinensis*	Identity%	e-value
		S1	S3	S5	S7						
Ciclev10031952m.g	*Ciclev10031952m*	1729.8	2878.0	2680.9	105.0	0.6	0.6	0.3	orange1.1t04194.2	98.6	0
Ciclev10031951m.g	*Ciclev10031951m*	614.3	1026.2	1329.9	220.8	0.9	0.8	0.7	orange1.1t04194.2	94.1	0
Ciclev10026344m.g	***Ciclev10026344m***	641.1	605.5	970.9	933.4	0.5	0.5	0.7	Cs1g22450.2	96.8	0
Ciclev10001661m.g	*Ciclev10001661m*	1614.2	7.9	0.4	0.1	-0.6	-0.3	-0.5	orange1.1t04194.2	85.6	0
Ciclev10029158m.g	*Ciclev10029158m*	165.5	304.9	335.8	268.7	0.9	0.6	0.7	Cs8g05410.1	98.0	0
Ciclev10005279m.g	*Ciclev10005279m*	90.3	182.7	405.2	158.8	1.0	0.9	1.0	Cs9g11480.1	96.6	0
Ciclev10031953m.g	*Ciclev10031953m*	111.5	187.1	168.8	12.8	0.6	0.6	0.3	Cs7g25580.1	99.7	0
Ciclev10009335m.g	*Ciclev10009335m*	109.4	93.7	75.7	68.5	-0.4	-0.2	-0.6	Cs4g13440.5	99.6	7.12E-179
Ciclev10005276m.g	*Ciclev10005276m*	31.6	76.5	174.8	60.4	1.0	0.9	1.0	Cs9g11480.1	96.6	0
Ciclev10012033m.g	*Ciclev10012033m*	4.7	78.3	111.4	67.1	0.9	0.6	0.8	Cs6g03020.1	99.7	0
Ciclev10005275m.g	*Ciclev10005275m*	24.4	47.8	118.3	56.3	0.9	0.9	1.0	Cs9g11480.1	96.4	0
Ciclev10020814m.g	*Ciclev10020814m*	41.8	36.3	61.2	53.5	0.6	0.6	0.8	Cs5g16290.1	95.9	0
Ciclev10012561m.g	*Ciclev10012561m*	8.3	26.1	24.8	131.3	-0.3	-0.4	-0.1	Cs6g04150.1	99.2	0
Ciclev10020828m.g	*Ciclev10020828m*	5.1	18.9	34.5	94.9	-0.1	-0.2	0.1	Cs5g24950.1	100.0	0
Ciclev10005297m.g	*Ciclev10005297m*	41.2	48.5	22.0	19.5	-0.3	-0.3	-0.6	Cs5g35100.1	97.5	0
Ciclev10015723m.g	*Ciclev10015723m*	25.1	22.6	37.9	32.3	0.6	0.7	0.9	Cs2g10090.1	98.6	0
Ciclev10020377m.g	*Ciclev10020377m*	1.2	7.5	21.1	33.0	0.2	0.1	0.4	Cs5g24940.1	99.7	0
Ciclev10015630m.g	*Ciclev10015630m*	13.6	17.9	11.9	15.0	-0.3	-0.6	-0.6	Cs2g16070.1	97.6	0
Ciclev10015724m.g	*Ciclev10015724m*	42.7	3.5	0.9	2.0	-0.6	-0.3	-0.5	Cs2g05400.1	97.8	0
Ciclev10009342m.g	*Ciclev10009342m*	14.3	9.6	12.7	12.3	-0.3	0.1	0.1	Cs4g13430.1	97.9	2.22E-175
Ciclev10020874m.g	*Ciclev10020874m*	0.4	27.6	6.0	0.9	0.3	0.0	-0.1	Cs5g16860.1	97.2	0
Ciclev10026347m.g	*Ciclev10026347m*	3.3	12.7	7.0	6.3	0.4	0.0	0.0	Cs1g12670.1	97.2	0
Ciclev10033543m.g	*Ciclev10033543m*	3.4	7.7	5.4	3.4	0.5	0.3	0.1	Cs7g25540.1	99.7	0
Ciclev10021579m.g	*Ciclev10021579m*	3.3	3.6	2.6	3.9	-0.8	-1.0	-0.8	orange1.1t00604.3	99.6	0
Ciclev10020875m.g	*Ciclev10020875m*	6.9	2.2	1.8	1.0	-0.5	-0.2	-0.5	Cs5g18050.1	98.0	0
Ciclev10002505m.g	*Ciclev10002505m*	2.1	4.7	2.7	2.0	0.3	0.0	-0.1	orange1.1t05216.1	96.0	4.07E-127
Ciclev10020873m.g	*Ciclev10020873m*	0.4	0.5	3.6	5.2	0.2	0.1	0.5	Cs5g18010.1	99.2	0
Ciclev10020870m.g	*Ciclev10020870m*	0.4	0.6	3.3	3.7	0.4	0.3	0.7	Cs5g18010.1	99.2	0
Ciclev10005230m.g	*Ciclev10005230m*	0.0	2.2	1.9	3.6	0.2	-0.2	0.2	Cs5g13560.1	89.8	0
Ciclev10005348m.g	*Ciclev10005348m*	0.6	1.2	1.6	0.3	0.9	0.8	0.7	Cs9g11480.1	91.9	0
Ciclev10006646m.g	*Ciclev10006646m*	3.2	0.1	0.1	0.0	-0.6	-0.2	-0.5	Cs9g11440.1	91.4	0
Ciclev10006423m.g	*Ciclev10006423m*	1.0	0.9	0.9	0.1	0.3	0.5	0.1	Cs9g11440.1	86.8	0
Ciclev10001555m.g	*Ciclev10001555m*	1.9	0.0	0.0	0.0	-0.6	-0.3	-0.5	Cs3g20650.1	95.8	0
Ciclev10015708m.g	*Ciclev10015708m*	0.4	0.1	1.3	0.0	0.8	1.0	0.9	Cs2g16040.1	98.1	0
Ciclev10020880m.g	*Ciclev10020880m*	0.1	0.2	0.5	0.7	0.3	0.1	0.5	orange1.1t04506.1	98.3	0
Ciclev10006704m.g	*Ciclev10006704m*	0.2	0.3	0.8	0.2	0.9	1.0	1.0	Cs5g35060.1	98.9	0
Ciclev10010561m.g	*Ciclev10010561m*	0.2	0.4	0.3	0.2	0.5	0.3	0.1	Cs7g25540.1	92.4	0
Ciclev10006550m.g	*Ciclev10006550m*	0.6	0.1	0.3	0.1	-0.3	0.1	-0.2	Cs9g11440.1	93.6	0
Ciclev10029876m.g	*Ciclev10029876m*	0.1	0.3	0.1	0.1	0.3	0.0	-0.1	Cs7g25540.1	91.3	0
Ciclev10010158m.g	*Ciclev10010158m*	0.1	0.2	0.1	0.1	0.2	0.0	-0.2	Cs7g25540.1	90.7	1.14E-128
Ciclev10015685m.g	*Ciclev10015685m*	0.2	0.1	0.3	0.0	0.6	0.8	0.6	orange1.1t02091.1	98.6	0
Ciclev10023568m.g	*Ciclev10023568m*	0.0	0.1	0.2	0.1	0.7	0.6	0.8	Cs5g17970.1	98.0	0
Ciclev10028731m.g	*Ciclev10028731m*	0.0	0.0	0.0	0.4	-0.4	-0.6	-0.2	Cs8g02130.1	99.7	0
Ciclev10029909m.g	*Ciclev10029909m*	0.2	0.0	0.0	0.1	-1.0	-0.8	-0.8	orange1.1t01353.1	65.7	6.63E-160
Ciclev10012087m.g	*Ciclev10012087m*	0.2	0.1	0.0	0.0	-0.6	-0.3	-0.6	Cs6g15390.1	98.6	0
Ciclev10023994m.g	*Ciclev10023994m*	0.0	0.1	0.0	0.1	-0.1	-0.4	-0.1	orange1.1t04506.1	94.3	0
Ciclev10027189m.g	*Ciclev10027189m*	0.0	0.1	0.1	0.0	0.7	0.4	0.4	Cs7g25540.1	80.5	1.63E-155
Ciclev10015705m.g	*Ciclev10015705m*	0.2	0.1	0.0	0.0	-0.6	-0.4	-0.7	Cs2g16040.1	95.3	0
Ciclev10017683m.g	*Ciclev10017683m*	0.0	0.0	0.1	0.0	1.0	0.9	1.0	orange1.1t02091.1	98.6	0
Ciclev10015762m.g	*Ciclev10015762m*	0.0	0.0	0.0	0.0	0.5	0.1	0.3	Cs2g16050.1	87.9	0
Ciclev10005309m.g	*Ciclev10005309m*	0.0	0.0	0.0	0.0	0.5	0.3	0.1	Cs5g35050.1	98.6	0
Ciclev10017649m.g	*Ciclev10017649m*	0.0	0.0	0.0	0.0	0.1	0.4	0.0	orange1.1t02091.1	93.2	0
Ciclev10017559m.g	*Ciclev10017559m*	0.1	0.0	0.0	0.0	-0.6	-0.3	-0.5	Cs2g05430.1	88.5	0
Ciclev10013393m.g	*Ciclev10013393m*	0.0	0.1	0.0	0.0	0.1	-0.2	-0.3	Cs6g03020.1	74.3	2.67E-170
Ciclev10018226m.g	*Ciclev10018226m*	0.0	0.1	0.0	0.0	0.1	-0.2	-0.3	orange1.1t02085.1	97.3	0

FPKM values of *OMT* genes and the correlation coefficients (Person *r*) between the transcript levels and PMF contents are described. The most identical peptide for each protein sequence of OMT in *Citrus sinensis* genome are also listed. Search and identity calculation were performed using local BLASTP against the *Citrus sinensis* polypeptides, with an *e* value cutoff of 1e-5. *CrOMT1* (*Ciclev10026344m*), indicated in blue font. The nine *OMT* genes fulfilled the above criteria, indicated in yellow shading. Members identified as flavonoid-related OMTs (Liu *et al.*, 2016), indicated in red font.

Table S4. Percentage amino acid identity shared by PFOMTs described in the phylogenetic tree.

**Identity%**	Ciclev10026344m	VpOMT4	VpOMT5	SlAnthOMT	VvAOMT	OsOMT17	OsOMT15	McPFOMT	ObPFOMT-1	AtCCoAOMT7
Ciclev10026344m	100.0									
VpOMT4	52.6	100.0								
VpOMT5	39.3	39.8	100.0							
SlAnthOMT	58.3	55.5	37.4	100.0						
VvAOMT	54.6	53.4	39.2	66.8	100.0					
OsOMT17	55.8	49.3	41.4	50.7	50.2	100.0				
OsOMT15	57.0	49.2	42.7	53.0	50.9	55.1	100.0			
McPFOMT	59.5	55.3	37.7	58.6	56.6	54.0	52.2	100.0		
ObPFOMT-1	60.2	54.4	39.5	56.4	48.9	56.0	51.9	61.3	100.0	
AtCCoAOMT7	58.4	53.1	39.2	55.5	52.1	57.1	57.6	62.5	63.8	100.0

Table S5. Accumulation of the three most abundant PMFs in two different tissues during 'Ougan' fruit maturation.

Tissue	Stage	Nobiletin mean ±SE	Tangeretin mean ±SE	5-HPMF mean ±SE
Flavedo	S1	1.28±0.07	0.92±0.04	0.37±0.01
	S2	0.93±0.06	0.54±0.05	0.21±0.01
	S3	1.87±0.13	0.95±0.05	0.41±0.04
	S4	1.85±0.21	0.92±0.07	0.44±0.03
	S5	2.55±0.06	1.46±0.04	0.66±0.03
	S6	1.95±0.17	1.12±0.07	0.48±0.05
	S7	1.48±0.04	0.82±0.03	0.44±0.02
	S8	1.25±0.02	0.73±0.02	0.24±0.01
Albedo	S5	0.20±0.06	0.11±0.04	0.06±0.01
	S6	0.16±0.02	0.08±0.01	0.05±0.00
	S7	0.09±0.01	0.05±0.01	ND
	S8	0.04±0.00	0.02±0.00	ND

Values are means ±SE (*n*=4) and represent mg per gram of fresh tissue weight (mg g^{-1} FW). ND, not detected.
